# Comparative Energy and Thermal Performance of Biomimetic Louver Retrofits in Hot-Arid Climate Residential Buildings

**DOI:** 10.3390/biomimetics11100688

**Published:** 2026-09-23

**Authors:** Sherouk Hamdy Seif, Mohamed Fikry, Tarek Farghaly

**Affiliations:** 1 Faculty of Engineering, Alexandria University, Alexandria 21544, Egypt; mohamed.ferky@alexu.edu.eg (M.F.); tarek.farghaly@alexu.edu.eg (T.F.); 2 Faculty of Energy and Environmental Engineering, The British University in Egypt, El Sherouk City 11837, Egypt

**Keywords:** biomimicry, sustainable materials, bio-inspired envelope retrofits, thermal performance, passive cooling, louvers

## Abstract

Building envelopes at this time of climate change should enhance their energy performance and, as a result, minimize undesirable environmental impacts. This study performed a comparative simulation analysis of a residential building in New Cairo, Egypt, to evaluate its performance following the implementation of a passive design element in relation to its traditional structure. The suggested passive design element consists of biomimetic louver shading devices manufactured from locally available natural and agricultural waste materials inspired by animal adaptation mechanisms in nature, including rice straw, rice husk, cotton waste fibers, palm fronds, jute fabric, and reflective aluminum foil, whereas the base-case scenario encompasses none of these. To accomplish this, the Energy Plus simulation engine was employed to conduct Design Builder simulations, and Meteonorm was employed to generate meteorological data for the site. Results indicate that biomimetic retrofit strategies achieved reductions in peak operative temperatures of 2.2–3.65 °C, improved thermal comfort conditions during summer months, and achieved cooling energy savings of 26–27% compared to the base case.

## 1. Introduction

The construction industry accounts for one-third of greenhouse gas emissions and more than 40% of worldwide energy consumption [1], making it critical for environmental initiatives, particularly in hot, arid countries [2]. Cairo, with a population of 20 million, has the challenge of extreme summer temperatures exceeding 40 °C [3], which are compounded by urban heat islands and reliance on energy-intensive air conditioning. Conventional construction methods prioritize short-term costs over efficiency [4], leading to poor thermal insulation and ventilation, which leads to excessive cooling costs and thermal discomfort for occupants [5].

With rising energy demand, pollution, and urban heat stress, the world is under more pressure. The danger is greatest in hot, dry places [6]. The residential sector is the biggest part of the building stock in Egypt and accounts for about half of the country’s electricity consumption. Recent estimates claim that households alone consume about 40–58% of the country’s electricity [7]. Much of this housing was built with little consideration for how it would deal with heat or energy efficiency. It relies primarily on air conditioning and other mechanical systems for cooling, which has become increasingly problematic as summers become hotter and longer [8]. This dependency not only causes the national grid to work harder and consume more electricity, contributing to greenhouse gas emissions, but it also puts a lot of strain on the national grid [9]. Because most of these buildings are already built and occupied, retrofitting them is more critical and difficult than constructing new energy-efficient buildings [10]. Thus, retrofitting is a critical goal, particularly in densely populated cities where housing demand and environmental concerns are increasing [11]. Predictions indicate that Egypt’s average annual temperatures would rise by 2 to 3 °C by 2050, particularly in inland locations such as Greater Cairo [12]. Conversely, the amount of rain that falls on the shore could be reduced by 7%.

These modifications are in keeping with the IPCC scenarios that predict significant greenhouse gas emissions, including SRES A2 [13]. These emissions will influence climate models for the future. The climate data used in this study shows that there is already a hot-dry regime, with summer temperatures often going above 40 °C [14], low humidity, and high solar radiation affecting structures that face south as shown in Figure 1. 

Egypt is very vulnerable to climate change on a national level [14]. It is already experiencing more heatwaves, heavy rain, and rising sea levels, even though it only contributes a small amount (less than 1%) to global greenhouse gas emissions [15]. National assessments and Egypt’s vision for the year 2050 [16] indicate that there is an urgent need to improve the energy efficiency and thermal resilience of the residential buildings in areas like Greater Cairo and New Cairo given the rising temperatures, water stress, and urban heat threats [17].

**Figure 1:**
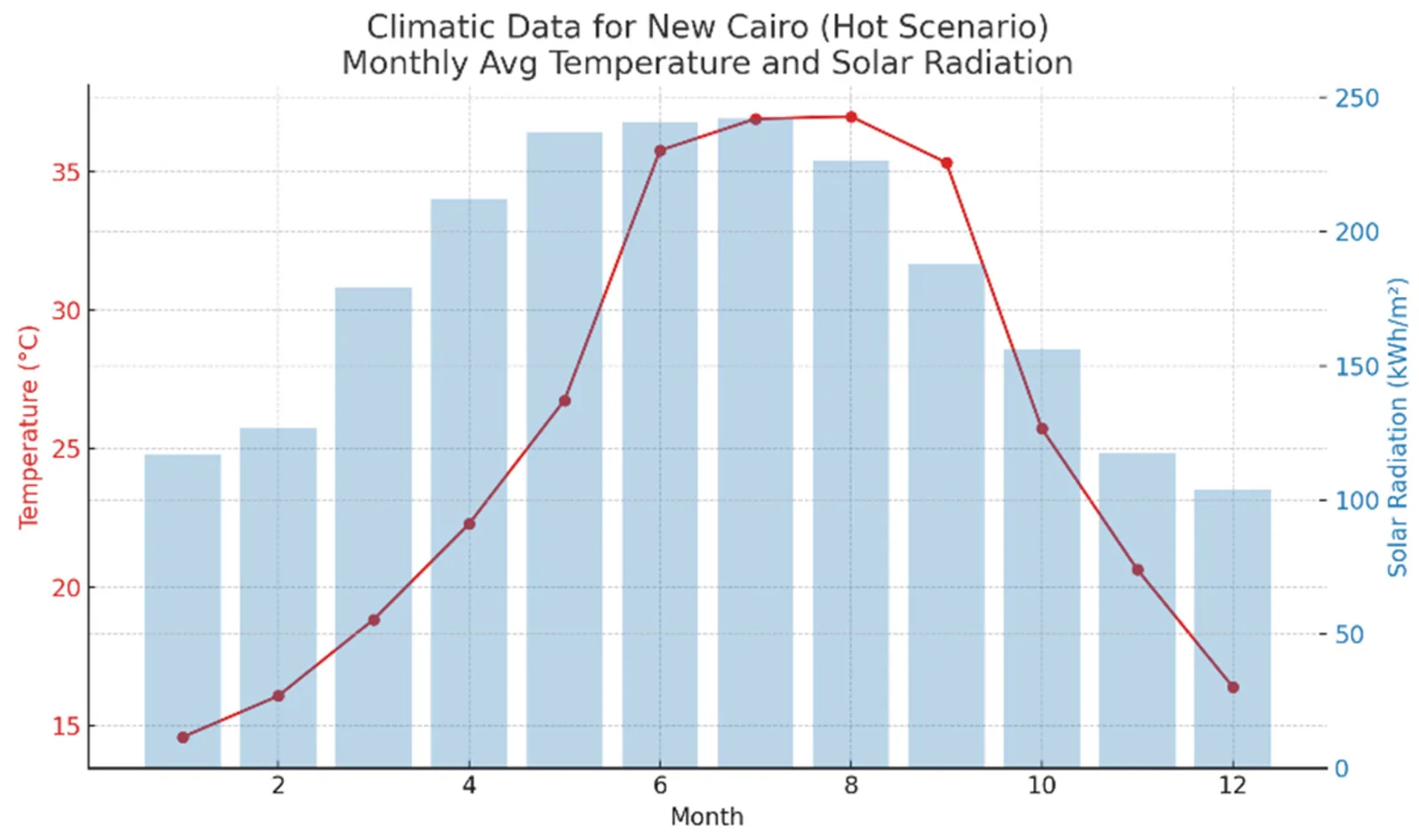
Climatic characteristics of New Cairo: monthly average dry-bulb temperature and global horizontal solar radiation.

The building envelope and its thermal insulation are advised to mitigate heat gain and, consequently, energy consumption. The building envelope regulates heat transfer between exterior and interior environments [18]; thus, an optimized envelope design can enhance thermal performance using passive solar strategies, making materials integral to this process [19].

Recently, biomimicry has emerged as a promising approach for enhancing building performance by learning from nature’s adaptive strategies [20]. Organisms living in hot and arid climates have developed efficient mechanisms to regulate heat transfer, reduce solar exposure [21], and maintain thermal balance under extreme climatic conditions [22]. These biological adaptations help to develop passive building solutions that enhance thermal performance and reduce energy demand [23]. 

Nature, however, was and has always been the first and most lasting teacher to humanity, and it has not been in vain since it has offered vast numbers of solutions that have been shaped by millions of years of adaptation to the harsh environment [24]. Desert and other hot-arid ecosystem organisms have worked out survival mechanisms, including thermal regulation, reflective covers, natural shading, and water retention, which are directly analogous to the tasks of buildings in the same climate [25]. Architects and engineers can learn and emulate these biological strategies to come up with viable and creative innovations for retrofitting. In this context, biomimicry can be an interface between biology and architecture, and it can help to design functional, sustainable, affordable, and fit local conditions [26].

One of the major challenges to life on Earth is extreme heat. Temperatures in the desert can pass 50 °C (122 °F), and the ground can reach 70 °C (158 °F). But incredible animals such as the fennec fox, the Arabian camel, the Saharan silver ant, and the thorny devil have evolved complex physiological, behavioral, and structural adaptations to survive these deadly conditions [27]. Many studies report these biological mechanisms; however, there is a pioneering gap in the translation of these natural strategies into practical, sustainable building materials, particularly using available agricultural waste locally in Egypt and similar regions [28].

In this study a function-oriented biomimetic approach is applied where biological thermoregulatory strategies are translated into engineering design principles for passive building envelopes. The agricultural waste materials selected are not to be a direct mimic of biological structures or tissues but to provide similar thermal functions as the biological strategies. 

The paper presents a novel biomimetic framework bridging two previously disparate fields: (1) thermoregulation strategies in desert animals and (2) six sustainable agricultural materials (rice straw, corn husk, cotton fibers, palm fronds, jute fabric, and reflective aluminum foils). We have a new approach where some animal adaptations are directly connected to material properties instead of the standard insulation methods that use synthetic materials or are energy-intensive to create. For instance, the 10× reflective hairs of the Saharan Silver Ant inspire our layer of aluminum foil, and the burrow microclimate (20–30 °C cooler) of the Fennec Fox inspires our rice straw insulation design, and the skin microchannels of the Thorny Devil inspire our palm frond layering strategy. 

This work has three main contributions: First, we construct the first systematic biomimetic mapping table that relates six heat-adapted animals to six specific agricultural materials with quantified thermal conductivity data. Second, we demonstrate that agricultural waste materials—typically discarded—can outperform conventional insulation while being carbon-negative, locally sourced, and cost-effective when used in outer louvers on buildings. Third, we introduce a triple-layer biomimetic system inspired by the combined strategies of camels, ants, and fennec foxes that combines evaporative cooling (cotton), radiative reflection (aluminum), and porous air trapping (rice/palm/corn husk), transforming waste into high-performance heat-resistant infrastructure—providing a sustainable approach to climate-resilient architecture in hot climates.

Corn husks and rice straw are both agricultural wastes in large quantities in Egypt, and they are usually burnt in open fields, which adds to the serious air pollution, as shown in Figure 2, when the authors visited a site in rural Egypt while rice straw and corn husks were being burned. Reusing such waste to insulate building spaces thus not only enhances the performance of buildings but also promotes sustainable waste management and the concept of a circular economy. Moreover, the hybrid system is representative of scalable and inexpensive bio-insulation that may be produced with ease using local resources and rudimentary pressing methods. A broader implementation of it would go a long way in supporting the national energy efficiency and carbon reduction goals of Egypt [29] in the context of Vision 2030 [30], as sustainable building design is in line with valorization of agriculture. The Egypt Vision 2030 Agenda [30] strongly addresses and supports the main direction of the present research, particularly through its emphasis on sustainable development, energy efficiency, environmental sustainability [31], and improving the performance of the built environment [32].

Unlike conventional shading systems, which primarily focus on reducing solar heat gain, the proposed biomimetic louver system simultaneously addresses an additional environmental challenge by utilizing locally available agricultural residues that are commonly disposed of through open-field burning in rural Egypt. Consequently, the proposed approach combines passive solar shading with agricultural waste valorization, providing a low-cost retrofit strategy that enhances building thermal performance while reducing air pollution and supporting circular economy principles. Accordingly, the objective of this study was to compare alternative agricultural waste materials within the same louver configuration rather than to evaluate different shading technologies.

This research aims to address the way in which biomimetic solutions based on the adaptive behaviors of organisms living in hot-arid environments can be used to inform building envelope retrofits to enhance thermal comfort, energy needs, and environmental implications. Using the lessons learned in natural systems together with cost-effective retrofitting methods, the research paper illustrates how localized solutions of little complexity can be investigated on a large scale to adjust performance throughout the residential sector in Egypt. Besides, these solutions will help in solving other environmental challenges that are not related to energy, such as decreasing air pollution, by reusing agricultural waste materials such as rice husks and palm fronds instead of burning them.

The research uses a mixed theory-quantitative comparative research design. The theoretical background starts with a literature-based theory on how biomimicry can be elaborated as guiding solutions in the design of building envelope sustainability. According to the solutions from nature’s inspirations, the study utilizes quantitative simulation modeling, which is based on climate data reflecting the conditions in the country in both current and future hot-arid seasons in Egypt (e.g., Typical Meteorological Year datasets) when examining nature-inspired envelope retrofit scenarios on exterior walls and windows of residential buildings. The model incorporates dynamic simulations of energy to examine the contribution of each of the retrofit interventions to the operative temperatures, energy demand, and comfort performance. The methodology will allow balancing the advantages and disadvantages of every intervention by providing concept validation and empirical evaluation because the approach integrates biological inspiration and performance-based analysis. This stringent approach enlightens scalability strategies to transform ecological methods of adapting to evidence-based cost-effective retrofit actions to the Egyptian housing sector.

The case study focuses on a residential building in New Cairo. This building was chosen because it is typical of the kinds of homes that already exist in New Cairo, Egypt’s hot, dry climate. The case study is a residential building in New Cairo. The building was chosen for its representative character of the prevailing residential building typology in New Cairo, Egypt’s hot, arid climate. The paper investigates biomimetic louver retrofits with reflective foils, natural fibers, and hybrid material configurations. The research compares the recommended retrofit techniques in terms of thermal comfort, cooling energy performance, and environmental advantages. The current assessment aims to find the best-performing biomimetic louver materials for improving building thermal performance and reducing cooling energy consumption in hot, arid climates. 

Based on these perspectives, the project will provide a new louver retrofit option for residential structures using biomimetic and nature-inspired materials. The suggested louver systems aim to improve façade shading efficacy, reduce solar heat gain, and increase inside thermal comfort while reducing cooling energy consumption. Furthermore, the various biomimetic louver material selections were compared to the basic case model to determine their capacity to minimize energy consumption while improving thermal comfort in residential structures.

## 2. Background

The majority of Egypt’s residential buildings are made of reinforced-concrete skeletons with uninsulated masonry infill walls and single-glazed openings [33]. As a result, they perform poorly under hot-arid climatic conditions and rely heavily on mechanical cooling. Recent studies show that the residential sector accounts for a substantial share of the country’s electricity consumption [15]. As summer temperatures rise and urban heat stress increases, the amount of electricity used for cooling is also growing [34]. In numerous rapidly developing regions, such as New Cairo, the majority of existing residences were constructed with little consideration for their adaptability to temperature fluctuations [35]. This makes large-scale retrofitting a top priority for lowering peak electricity demand, greenhouse gas emissions, and stress on the national grid [36].

Despite this growing body of research, the literature on biomimetic applications remains strongly skewed towards new landmark or experimental buildings, where advanced façade technologies and novel materials are tested in high-profile projects [35]. Reviews of biomimetic and adaptive envelopes in hot and dry climates report that most proposals are conceptual frameworks, hypothetical models or small-scale prototypes evaluated through digital simulations, often for office or iconic public buildings rather than ordinary housing [37]. In contrast, there is very limited empirical or simulation-based evidence on how biomimetic strategies can be translated into retrofit solutions for existing residential building stocks in hot-arid contexts such as Egypt, where simple, low-maintenance, and low-cost interventions are urgently needed [38].

Furthermore, several gaps can be identified regarding building type, climate context, modeling approach, and scalability. First, the majority of biomimetic case studies focus on bespoke or high-end developments, with little attention to the widespread, medium-income apartment blocks that dominate the Egyptian residential sector [39]. Second, many applications are documented in temperate or generic warm climates, while only a small subset explicitly addresses the extreme summer conditions, high solar gains, and low diurnal temperature swings typical of hot-arid regions [40]. Third, while many studies examine individual biomimetic concepts, comparative simulations of multiple envelope strategies within a uniform methodological framework are limited, hindering the prioritization of solutions for extensive retrofits. Lastly, there is still not much guidance on how to scale up biomimetic measures from individual buildings to neighborhoods or cities, taking into account cost, constructability, and the availability of materials in the area [41]. Table 1 summarizes the main research gaps identified in the literature.

In addition to these knowledge gaps, practical barriers hinder the implementation of biomimetic retrofits in hot-arid housing [40]. Many documented biomimetic strategies are technologically complex and rely on advanced technologies, making them difficult to implement in ordinary residential neighborhoods where budgets, technical expertise, and maintenance resources are limited [39]. Additionally, only a limited number of studies explicitly investigate the performance of biomimetic envelopes under the extreme summer conditions typical of Egyptian cities, where extensive air-conditioning usage intensifies grid stress and emissions [42].

These research and implementation gaps provide the foundation for the biomimetic material selection and comparative evaluation framework presented in the following sections.

## 3. Biomimetic Heat-Resistant Insulation: From Desert Animal Adaptations to Sustainable Agricultural Material

Biomimetic heat-resistant insulation is based on desert creatures’ adaptation systems for controlling heat flow and maintaining interior temperatures in severe climate conditions. These natural strategies have inspired the development of sustainable building materials with improved thermal insulation performance and lower environmental impact [43]. Agricultural residues such as rice husks, corn residues, and palm-based materials have received increasing attention as renewable bio-based insulation materials [44], due to their low thermal conductivity, low embodied carbon, biodegradability, and abundance in nature [45]. The combination of biomimetic concepts and agricultural waste valorization provides an environmentally responsible way to improve building thermal performance [46], while promoting circular economy principles and lowering construction-related environmental consequences [47].

### 3.1. Reflective Aluminum Foil Inspired by the Silver Ant of the Sahara

The Sahara ant (*Cataglyphis bombycina*) survives the extreme desert heat thanks to special silvery hairs that are ten times more reflective than the hairs of shaved ants. The triangular hairs behave like microscopic mirrors, reflecting over 90% of the visible and near-infrared solar radiation through total internal reflection [48,49,50]. This technique biomimetically resembles reflective aluminum foil with a mirror-like surface that reflects more than 95% of incoming solar radiation [51]. Aluminum also functions as a radiant barrier, reflecting thermal energy before it is absorbed into the building envelope, limiting heat retention and improving overall thermal performance by reducing radiant heat transfer [52].

### 3.2. Rice Straw and Corn Husk Inspired by Fennec Fox Burrows and Camel Thermoregulation

The Fennec fox maintains thermal comfort by digging underground burrows, which are about 20–30 °C cooler than the desert surface [51]. This natural way is similar to the porous structure of rice straw and rice husk, where the interconnected fibers trap air and create an effective thermal barrier, which reduces the conductive heat transfer [53,54,55]. Similarly, the camel can store heat during the day and slowly release it at night to avoid rapid temperature fluctuations [56]. Rice straw has a low thermal conductivity of around 0.051–0.053 W/m.K and rice husk has a thermal conductivity of around 0.08 W/m.K, both of which resist heat transfer and provide thermal buffering [57,58,59,60]. The presence of many microscopic pores increases the insulation by increasing the trapped air inside the material structure [61,62].

### 3.3. Palm Fronds Inspired by Thorny Devil Skin and Desert Iguana Burrowing

Interlinked skin microchannels on the Thorny Devil (*Moloch horridus*) efficiently transport water across its body surface [63]. The biological water collection channels, which have an interconnected geometry similar to the layered fibrous structure of palm fronds [64], where the presence of multiple layers of fibers and internal air gaps improves the thermal insulation by trapping air [65], are where extreme heat can also be escaped by the desert iguana, which burrows into the stable thermal environment underground [66]. The thermal conductivity of palm fronds is between 0.047 and 0.070 W/m·K and shows excellent insulation performance in hot climate conditions [53]. Composite palm-frond systems further improve thermal performance in repeated heating and cooling cycles by keeping stable thermal resistance at different environmental temperatures [67].

### 3.4. Cotton Fibers Inspired by Red Kangaroo and Elephant Thermoregulation

Under hot conditions, the red kangaroo cools itself efficiently by evaporative cooling of saliva licking its forearms. This mechanism is similar to the highly porous and absorbent nature of cotton fibers [68], which provide moisture transport to enhance their evaporative cooling potential. In addition, elephants also use their large ears, which have many capillaries, to help control their body temperature by dissipating heat. Similarly, cotton fiber networks contain many air-filled pores that increase thermal resistance while allowing air flow, which contributes to passive cooling and improved thermal comfort [69].

### 3.5. Jute Fabric Inspired by the Kangaroo Rat and Sidewinder Snake

The kangaroo rat lives in dry habitats, and it makes very concentrated pee, so it conserves water and makes the best use of resources [70]. This adaptation is similar to that of jute fabric [71]. The porous constitution of the jute fabric’s natural fiber structure is responsible for effectively trapping air, thereby reducing thermal conductivity and restricting heat transfer through the material [72]. The sidewinder rattlesnake, by its characteristic side-winding gait, reduces the surface area of its body in contact with the hot desert sand and thus limits heat gain [73]. Moreover, the porous, interconnected network of jute fibers also allows for less conductive heat transfer due to the higher amount of trapped air inside the material, which results in better thermal insulation [73].

### 3.6. Summary of Biomimetic Mapping

The selected biomimetic materials demonstrate clear functional similarities with the thermoregulatory strategies of desert organisms. The reflective aluminum foil mimics the high solar reflectance of the Saharan silver ant, and the rice straw mimics the air-trapping insulation mechanism of the Fennec fox burrows. The corn husk mimics the thermal buffering strategy of the camel by delaying the heat transfer due to its low thermal conductivity. The palm fronds mimic the layered microchannel structure of the Thorny Devil and the stable thermal environment of the desert iguana burrows. The cotton waste fibers mimic the evaporative cooling strategy of the red kangaroo and the heat dissipation mechanism of the elephant ears. Finally, the jute fabric mimics the efficient thermal conservation of the kangaroo rat and the minimized heat gain of the sidewinder snake through the dense porous fiber network and low thermal conductivity, as shown in Figure 3.

## 4. Methodology

The main objective of this study is to evaluate the use of external louvers made from agricultural waste materials, inspired by the thermoregulatory strategies of desert animals, as a new shading method for building envelopes in the housing industry, particularly under future climate change conditions, compared to traditional single-glazed windows. A residential building was simulated in the climatic conditions of the New Cairo, Egypt, zone, which represents a hot desert climate (BWh) according to the Köppen–Geiger climate classification [74], with extremely low annual precipitation, high solar radiation, and prolonged hot summer conditions. 

A comparative analysis of a case study building in New Cairo, Egypt, was performed using simulations for both the as-built structure (base case) and the hypothetical passive design of the same building (modified case). This investigation evaluated the impact of passive design in diminishing energy usage and enhancing the thermal performance of the case study building. The methodology comprises two steps: (1) employing Meteonorm software (version 8.2) [75] and (2) conducting simulations using DesignBuilder software (version 7.3.1.003–educational edition) and EnergyPlus (version 9.4.0.002) as the simulation engine [76]. The overall research workflow is summarized in Figure 4.

### 4.1. Case Study

The selected case-study building is a detached residential building located in New Cairo, Egypt. It was selected as a representative example of the prevailing residential building typology in New Cairo, where residential buildings within communities (compounds) are typically detached, range from five to six stories in height, and are separated by landscaped open spaces. This urban configuration fully exposes all façades to the outdoor environment, preventing thermal interaction with adjacent buildings, as shown in Figure 5. Accordingly, the simulation model was developed using boundary conditions representative of this urban context, with all external façades exposed to ambient climatic conditions. The proposed biomimetic louver retrofit was applied to the south-facing façade, which receives the highest solar exposure during the cooling season. Although the investigation focuses on a single case-study building, it represents a common residential typology widely adopted in New Cairo, providing a realistic and relevant basis for evaluating the proposed retrofit strategy.

The selected case study is a representative residential building typology commonly found in New Cairo, Egypt. The gross floor area of the building is approximately 3800 m^2^, which comprises 5 to 6 floors, each divided into 4 apartments of different sizes. The average number of occupants per flat is four to five. Figure 6 displays an overview of the proposed residential building model. The base case consists of a single glazed window. The external glazing is made up of a single 6 mm thick clear glass panel. The overall thermal transmittance (U-value) for this external glazing is recorded at 5.778 W/m^2^K, whereas the modified case comprises the use of biomimetic louvers.

The case study was selected because its architectural layout, construction characteristics, and façade configuration are typical of many contemporary residential developments in the area. Therefore, while the quantitative results presented in this study are specific to the investigated building, the proposed methodology and the comparative assessment of the biomimetic retrofit strategies can provide valuable guidance for similar residential buildings operating under comparable hot-arid climatic conditions.

**Figure 6:**
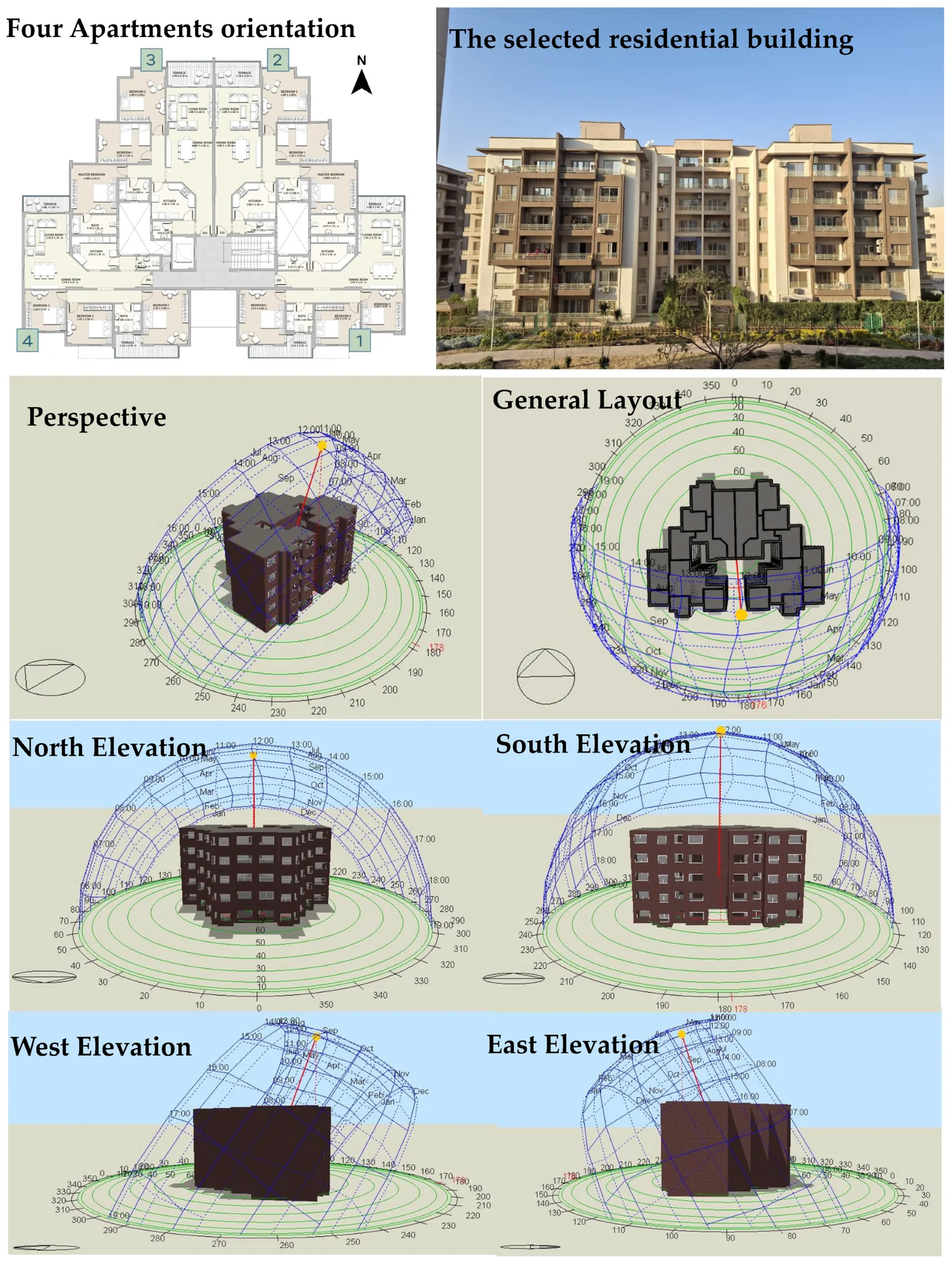
Proposed Residential Building Model Overview.

### 4.2. Using Meteonorm Software

The Meteonorm program [75] was utilized to obtain the actual climatic data for the New Cairo City area from weather data files pertinent to Egypt. The purpose of extracting the weather data is to acquire accurate climate information for this specific place instead of utilizing the overall climate data for Cairo City.

### 4.3. Energy and Thermal Performance Simulation

The thermal and energy performance of the building was evaluated for each proposed biomimetic louver material. Simulations were conducted during the summer months (June, July, August, and September). 

To provide a clearer representation of the climatic conditions used in the simulations, Figure 7 presents the monthly average and maximum outdoor air temperatures together with the global horizontal solar radiation during the analyzed summer period (June–September). The climatic data were obtained from the Meteonorm weather database and used as the weather input for the DesignBuilder/EnergyPlus simulations. August exhibited the highest maximum outdoor air temperature, while consistently high levels of solar radiation were recorded throughout the summer months. These climatic conditions justify the selection of the summer period for evaluating the thermal and energy performance of the proposed biomimetic façade retrofit strategies.

Using the base-case model, six window louver retrofit scenarios were simulated, as summarized in Table 2 and illustrated in Figure 8. Additional simulation input parameters and operational schedules adopted in the Design Builder/Energy Plus model are provided in Appendix A to facilitate model transparency and reproducibility.

The objective was to ascertain the most efficient scenario for minimizing energy use and CO_2_ emissions in New Cairo’s hot-arid climate, taking into account performance metrics such as solar heat gains and cooling energy consumption. Appendix A delineates the thermophysical characteristics of the suggested bio-based and hybrid retrofit louver materials. 

The conceptual design evolution of the suggested biomimetic louver system is depicted in the illustrations found in Appendix C. These illustrations depict the arrangement of louvers, solar shading approach, daylight management idea, hybrid material structure, and theoretical panel construction. They are incorporated to elucidate the design reasoning and showcase the conceptual viability of transforming agricultural waste into bio-based louver panels. The sketches are intended for conceptual illustration only, while the numerical simulations were performed using the simplified louver geometry implemented in Design Builder.

The proposed biomimetic louvers were conceptually modeled as bio-composite panels fabricated from agricultural waste fibers using conventional natural-fiber composite manufacturing techniques reported in the literature [77,78,79,80]. In general, the fabrication process involves cleaning and drying the agricultural residues, followed by grinding or fiber preparation, mixing the fibers with a suitable bio-based or polymeric binder, compression molding (hot pressing) to form rigid composite panels, and finally cutting the panels into louver blades of the required dimensions. The conceptual fabrication pathway is illustrated in Appendix C. It should be noted that the present study focuses on the thermal and energy performance evaluation of the proposed louver systems through numerical simulation, whereas the detailed manufacturing process is beyond the scope of this work.

**Figure 8:**
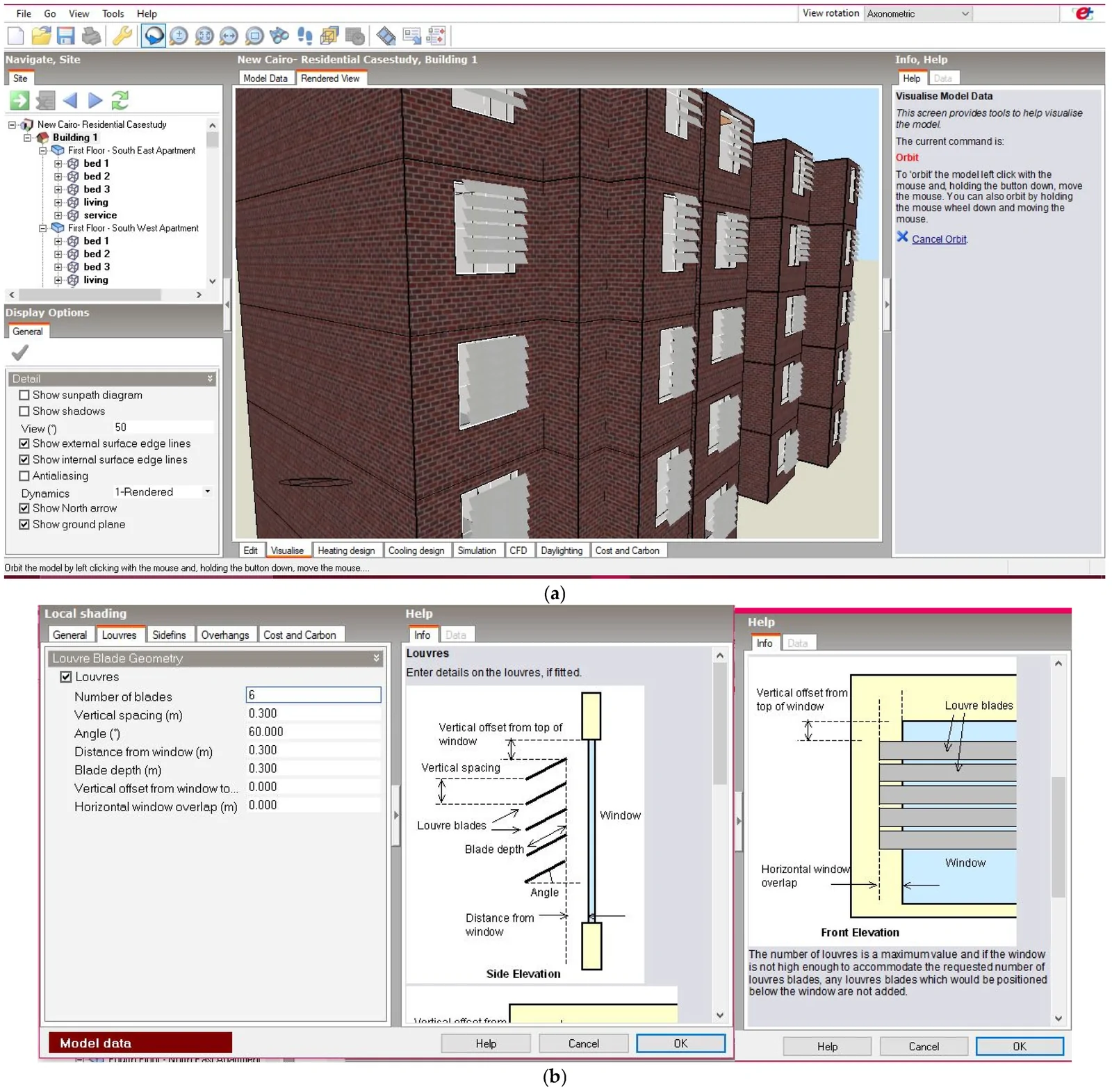
Design Builder model and louver configuration: (**a**) building model illustrating the installation of louvered shading devices on the façade; and (**b**) local shading settings and schematic illustrations of the louver geometry and positioning parameters used in the simulation.

The proposed biomimetic concept is not restricted to this geometry, despite the fact that a fixed horizontal louver configuration was implemented in the present study to facilitate consistent energy simulations in DesignBuilder. The system may also be implemented as operable sliding, folding, or perforated louver panels while maintaining the same biomimetic principles, depending on architectural and operational requirements (Appendix C).

The geometric configuration of the proposed louver system was determined by the design principles and recommendations for external shading devices that were reported in previous studies [81,82,83,84].

These studies identify blade inclination, spacing, depth, and the distance between the louver system and the window as critical parameters that influence thermal performance and solar control [85]. Accordingly, a single reference louver geometry was adopted and maintained throughout all simulation scenarios to ensure a fair comparison between the investigated biomimetic materials. As a result, the blade material was the sole variable in the comparative analysis, while all other geometric parameters, such as the number of blades, blade thickness, spacing, inclination angle, blade depth, and distance from the window, were maintained at a constant value.

The conceptual evolution of the proposed louver system is detailed in Appendix C, while Figure 8 illustrates the simplified geometry that was implemented for the numerical simulations.

**Table 2:** Residential Building Window Louver Retrofit Alternatives.

Blade Material	No. of Blades	Thickness (m)	Spacing (m)	Angle (°)	Depth (m)	Distance from Window (m)
Rice straw	6	0.012	0.300	60	0.300	0.300
Palm fronds	6	0.012	0.300	60	0.300	0.300
Jute fabric	6	0.012	0.300	60	0.300	0.300
Hybrid: Aluminum + Cotton waste fibers	6	0.012	0.300	60	0.300	0.300
Hybrid: Rice straw + Corn husk	6	0.012	0.300	60	0.300	0.300
Corn husk	6	0.012	0.300	60	0.300	0.300

The geometric dimensions of the louvers were selected based on commonly adopted external shading configurations reported in the literature [81,82,83,84,85] and were maintained constant throughout all simulation scenarios to ensure a fair comparison. Accordingly, blade thickness, spacing, inclination angle, projection depth, and distance from the glazing remained unchanged, while the blade material was considered the only variable, allowing the influence of material properties on thermal comfort and cooling energy performance to be evaluated independently of geometric effects.

To clarify the practical implementation of the proposed retrofit strategy, the louvers were conceptually designed to be installed independently in front of the existing windows using side-mounted supporting brackets, maintaining a 300 mm clearance between the glazing and the louver system. This arrangement allows unobstructed window operation, natural ventilation, cleaning, and maintenance. Although a fixed horizontal louver configuration was adopted for the numerical simulations to ensure consistent DesignBuilder analyses, the proposed biomimetic concept may also be adapted into operable configurations, such as sliding, folding, or perforated louver systems, according to architectural and functional requirements. Detailed installation concepts, cross-sectional sketches, and alternative louver configurations are provided in Appendix C.

The investigated agricultural waste materials were conceptually represented as bio-based composite louver panels fabricated using conventional natural-fiber composite manufacturing techniques reported in the literature. However, the present study focuses exclusively on evaluating the thermal and energy performance of these conceptual louver systems through numerical simulation; therefore, the detailed manufacturing process is beyond the scope of this work.

The bio-based alternatives (rice straw, palm fronds, jute fabric, and corn husk) were represented as monolithic compressed panels, whereas the aluminum + cotton waste fiber hybrid was represented as a layered composite panel comprising an external reflective aluminum layer and an internal cotton-waste insulation layer. The rice straw + corn husk hybrid was represented as a homogeneous composite panel. A conceptual illustration of the structural configurations of all proposed louver systems, together with the proposed fabrication pathway, is provided in Appendix C to further clarify the construction concept of the investigated retrofit alternatives.

## 5. Results

The simulation model generated a large number of graphical outputs, including contour plots for all investigated scenarios. To ensure clarity and conciseness, the most relevant quantitative findings were extracted and presented in Table 3, Table 4, Table 5, Table 6, Table 7, Table 8 and Table 9 and comparative charts throughout Section 5. The complete set of representative contour plots is provided in Appendix D to offer a visual interpretation of the thermal performance and support the reported findings.

The Design-Builder (Energy Plus) simulation outputs were directly used to derive the operative temperature values in this study [86]. Operative temperature was selected, as it is a more precise indicator of indoor thermal comfort than air temperature alone, as it accounts for both convective and radiative heat transfer between individuals and their surroundings. This is particularly relevant when evaluating external shading systems, as biomimetic louvers reduce the temperature of both indoor air and adjacent building surfaces, thereby improving the thermal comfort of occupants [87].

The sensible cooling load is the energy required to reduce the interior air temperature, whereas the total cooling load includes both sensible and latent cooling loads, as it reflects the building’s comprehensive cooling needs [86]. Design Builder automatically generated the performance outputs using the Energy Plus simulation engine after each simulation run. Operational temperature, relative humidity, solar heat gains through windows, cooling electricity consumption, perceptible cooling load, total cooling load, and operational CO_2_ emissions were all included in the simulation outputs. The baseline building and the investigated biomimetic louver retrofit alternatives were subsequently compared using these performance indicators (Appendix B).

The EnergyPlus simulation outputs provide a detailed visualization of the thermal and energy performance of the baseline building and the investigated biomimetic louver alternatives. The representative outputs in Appendix D are categorized into five groups for each scenario: (a) indoor comfort conditions, which include air temperature, radiant temperature, operative temperature, and outdoor dry-bulb temperature; (b) internal and solar heat gains, as well as the total latent load; (c) operational CO_2_ emissions; (d) sensible and total cooling loads; and (e) the monthly fuel breakdown, which includes room electricity, lighting, and cooling electricity consumption. This graphical output offers supplementary visual support for the quantitative values presented in Table 3, Table 4, Table 5, Table 6, Table 7, Table 8 and Table 9 and enables the examination of the summer monthly variation in thermal and energy performance for each retrofit scenario (Figure A7, Figure A8, Figure A9, Figure A10, Figure A11, Figure A12 and Figure A13). 

The EnergyPlus outputs for the base case indicate that indoor thermal conditions were comparatively stable from June to September, with operative temperatures remaining within a narrow range despite the variation in outdoor conditions (Figure A7a). Throughout the summer period, the baseline also demonstrates comparatively high solar and internal heat gains, as well as cooling-related burdens (Figure A7b,d). The quantitative results reported in Table 3 (Figure A7c,e) are consistent with the fact that the corresponding CO_2_ emissions and cooling electricity consumption increase during the peak summer months, reaching their highest values in August. 

In comparison to the base case, the rice-straw louver reduces the indoor operative temperature and the corresponding cooling demand (Figure A8a,d,e). The average reduction of 2.20 °C in operative temperature and 16.9% in cooling electricity reported in Table 10 is also supported by the monthly EnergyPlus outputs, which demonstrate a decrease in cooling-related energy consumption and CO_2_ emissions. 

The jute-fabric louver enhances thermal and energy efficacy more than the rice-straw configuration. The EnergyPlus outputs indicate a reduction in cooling burdens and lower operative temperatures during the summer months (Figure A9a,d). In accordance with the average operative-temperature reduction of 2.42 °C and the 22.2% reduction in cooling electricity reported in Table 10, the monthly outputs (Figure A9c,e) also demonstrate the corresponding reduction in cooling electricity and CO_2_ emissions. 

Among the alternatives that were examined, the aluminum–cotton waste hybrid louver exhibits one of the most significant enhancements. The EnergyPlus comfort outputs demonstrate a significant decrease in operative temperature compared to the base case (Figure A10a), whereas the system-load and fuel-breakdown results suggest a significant decrease in cooling demand and electricity consumption (Figure A10d,e). The average operative-temperature reduction of 3.52 °C and the 26.8% reduction in cooling electricity reported in Table 10 are corroborated by these graphical results. 

Similarly, the rice-straw–corn-husk hybrid louver results in the most significant average reduction in operative temperature among the configurations that were examined. Throughout the simulated summer period, the EnergyPlus outputs indicate a decrease in operative temperatures, as well as a decrease in cooling loads and cooling electricity consumption (Figure A11a,d,e). The results indicate a 26.3% decrease in cooling electricity and an average operative-temperature reduction of 3.65 °C in comparison to the basal case. 

The corn-husk louver also significantly reduces the need for refrigeration. The EnergyPlus outputs demonstrate enhanced indoor thermal conditions and decreased cooling-related loads in comparison to the baseline (Figure A12a,d), as well as decreased cooling electricity consumption and CO_2_ emissions (Figure A12c,e). These findings are in accordance with the 2.65 °C average decrease in operative temperature and the 24.9% decrease in cooling electricity that are detailed in Table 10. 

Lastly, the palm-frond louver exhibits a comparable overall trend, with lower operative temperatures, cooling loads, cooling electricity consumption, and CO_2_ emissions than the base case (Figure A13a–e). The quantitative results are visually confirmed by the EnergyPlus outputs, which indicate a 24.6% reduction in cooling electricity and an average operative-temperature reduction of 2.60 °C.

### 5.1. Base Case

The simulation results for the residential building during the summer period (June–September) showed that the HVAC system was able to maintain acceptable indoor thermal comfort conditions despite the harsh outdoor climate. The average indoor air temperature was between 24.5 °C and 24.8 °C, and the operative temperature was between 24.3 °C and 24.6 °C, even though the outdoor dry-bulb temperature reached 36.36 °C in August. The relative humidity was within the comfort limits of 48% to 50.5%.

The cooling demand increased significantly in the hottest months with high solar heat gains through the building envelope. Solar gains through exterior windows increased from 12,371 kWh in June to 13,102 kWh in August, which led to a corresponding increase in sensible cooling load from approximately 39,702 kWh in June to 43,012 kWh in August. Similarly, the total cooling load increased from about 46,000 kWh in June to nearly 50,000 kWh in August.

The fuel breakdown analysis indicated that the cooling electricity was the largest portion of the building energy consumption, rising from 10,300 kWh in June to a maximum of 11,200 kWh in August, while the room electricity and lighting consumption remained relatively stable at 3266–3375 kWh and 1754–1813 kWh, respectively. The increased cooling energy demand was reflected in the operational carbon emissions, which rose from 9305 kg CO_2_ in June to 10,017 kg CO_2_ in August, before dropping to 9062 kg CO_2_ in September. The detailed simulation results are presented in Table 3. Detailed monthly results for thermal comfort, cooling loads, internal and solar heat gains, fuel consumption, and CO_2_ emissions are taken from the design builder software. To facilitate comparison between the base case building and the proposed retrofit solutions, the main analysis focuses on two key performance indicators: cooling energy consumption and indoor operative temperature.

**Table 3:** Base case summer simulation results.

Indicator	Unit	Jun	Jul	Aug	Sep
Operative Temperature	°C	24.50	24.40	24.60	24.30
Relative Humidity	%	50.00	49.00	48.00	50.50
Solar Gains Through Windows	kWh	12,371.04	12,900.57	13,102.32	14,642.58
Cooling Electricity	kWh	10,300.00	10,700.00	11,200.00	10,000.00
Sensible Cooling Load	kWh	−39,701.84	−41,427.36	−43,012.48	−39,502.31
Total Cooling Load	kWh	−46,000.12	−48,000.65	−50,000.21	−45,000.37
CO_2_ Emission	kg	9305.45	9662.14	10,017.36	9061.77

### 5.2. Simulation Optimization Results

The simulation results for the investigated louver configurations and materials are summarized in Table 4, Table 5, Table 6, Table 7, Table 8 and Table 9. The results were evaluated in terms of energy consumption, cooling performance, thermal comfort, and environmental impact.

#### 5.2.1. Rice Straw Louvers

The detailed simulation results for rice straw louvers are presented in Table 4.

**Table 4:** Rice straw louvers: summer simulation results.

Indicator	Unit	Jun	Jul	Aug	Sep
Operative Temperature	°C	22.30	22.20	22.40	22.10
Relative Humidity	%	47.8	46.8	45.8	49.0
Solar Gains Through Windows	kWh	8050.12	8120.45	8320.35	8650.28
Cooling Electricity	kWh	8400.00	8700.00	9100.00	8850.00
Sensible Cooling Load	kWh	−25,000.18	−26,800.36	−27,900.42	−26,200.21
Total Cooling Load	kWh	−38,200.45	−40,100.52	−41,900.63	−39,500.34
CO_2_ Emission	kg	7650.12	7981.35	8247.63	8020.89

#### 5.2.2. Jute Fabric Louvers 

The detailed simulation results for jute fabric louvers are presented in Table 5.

**Table 5:** Jute Fabric Louvers summer simulation results.

Indicator	Unit	Jun	Jul	Aug	Sep
Operative Temperature	°C	22.10	22.00	22.20	21.80
Relative Humidity	%	47.00	46.00	44.50	48.50
Solar Gains Through Windows	kWh	7200.45	7425.30	7780.25	8150.30
Cooling Electricity	kWh	7950	8250	8650	8000
Sensible Cooling Load	kWh	−22,650.18	−24,200.36	−25,800.42	−23,200.21
Total Cooling Load	kWh	−32,500.45	−34,900.52	−36,900.63	−33,100.34
CO_2_ Emission	kg	7240	7520	7880	7290

#### 5.2.3. Aluminum & Cotton Waste Hybrid Louvers

The detailed simulation results for hybrid material louvers are presented in Table 6.

**Table 6:** Aluminum & Cotton Waste Hybrid Louvers summer simulation results.

Indicator	Unit	Jun	Jul	Aug	Sep
Operative Temperature	°C	21.10	20.90	21.00	20.70
Relative Humidity	%	45.00	44.00	43.00	47.50
Solar Gains Through Windows	kWh	6350.45	6520.32	6880.30	7150.28
Cooling Electricity	kWh	7500	7850	8250	7300
Sensible Cooling Load	kWh	−21,200.18	−22,900.36	−24,100.42	−21,000.21
Total Cooling Load	kWh	−30,400.45	−32,900.52	−34,600.63	−30,000.34
CO_2_ Emission	kg	6780.45	7150.32	7650.68	6580.21

#### 5.2.4. Rice Straw + Corn Husk Hybrid Louvers

The detailed simulation results for hybrid material louvers are presented in Table 7.

**Table 7:** Rice Straw + Corn Husk Hybrid Louvers Summer Simulation Results.

Indicator	Unit	Jun	Jul	Aug	Sep
Operative Temperature	°C	21.00	20.80	20.90	20.50
Relative Humidity	%	46.50	45.50	44.00	47.00
Solar Gains Through Windows	kWh	6100.50	6280.30	6650.45	6950.30
Cooling Electricity	kWh	7600.00	7900.00	8300.00	7300.00
Sensible Cooling Load	kWh	−19,250.18	−20,600.36	−21,900.42	−18,550.21
Total Cooling Load	kWh	−31,800.45	−33,500.52	−34,900.63	−30,100.34
CO_2_ Emission	kg	6420.35	6780.45	7080.62	6130.21

#### 5.2.5. Corn Husk Louvers

The detailed simulation results for louvers made from corn husk material are presented in Table 8.

**Table 8:** Corn Husk Louvers summer simulation results.

Indicator	Unit	Jun	Jul	Aug	Sep
Operative Temperature	°C	21.95	21.75	21.90	21.60
Relative Humidity	%	45.00	44.00	43.00	46.50
Solar Gains Through Windows	kWh	6680.45	6901.35	7212.30	7650.30
Cooling Electricity	kWh	7700.00	8000.00	8450.00	7550.00
Sensible Cooling Load	kWh	−18,950.18	−20,950.28	−22,230.58	−18,130.21
Total Cooling Load	kWh	−25,300.45	−27,900.52	−29,600.63	−24,300.34
CO_2_ Emission	kg	6480.35	6750.35	7120.45	6360.20

#### 5.2.6. Palm Frond Louvers

The detailed simulation results for hybrid material louvers are presented in Table 9.

**Table 9:** Palm frond louvers’ summer simulation results.

Indicator	Unit	Jun	Jul	Aug	Sep
Operative Temperature	°C	21.85	21.65	21.80	21.50
Relative Humidity	%	45.50	44.50	43.50	46.80
Solar Gains Through Windows	kWh	6520.30	6760.20	7050.15	7480.40
Cooling Electricity	kWh	7650.00	7950.00	8350.00	7450.00
Sensible Cooling Load	kWh	−18,600.25	−20,550.40	−21,900.35	−17,900.50
Total Cooling Load	kWh	−24,900.60	−27,450.30	−29,150.80	−23,950.70
CO_2_ Emission	kg	6430.20	6710.50	7050.40	6280.30

### 5.3. Comparison

The comparative results are summarized in Table 10 and indicate that the hybrid agricultural waste louvers demonstrated the highest overall thermal performance, achieving up to a 3.65 °C reduction in operative temperature and approximately a 26–27% reduction in cooling electricity compared to the base case. The comparison presented in Figure 9. And Figure 10 shows that all bio-inspired louver materials reduced cooling electricity consumption and operative temp relative to the base case. 

The average values presented in Table 10 were obtained from the monthly simulation outputs, while the percentage reductions were calculated by comparing each retrofit scenario with the base case.

**Table 10:** Comparative performance between the base case and the bio-inspired louver materials.

Material	Average Operative Temp (°C)	Difference from Base Case (°C) ↓	Average Cooling Electricity (kWh) ↓	Difference from Base Case (%) ↓
Base Case	24.45	—	10,550	—
Rice Straw	22.25	−2.20	8763	−16.9%
Jute Fabric	22.03	−2.42	8213	−22.2%
Aluminum + Cotton Waste Hybrid	20.93	−3.52	7725	−26.8%
Rice Straw + Corn Husk Hybrid	20.80	−3.65	7775	−26.3%
Corn Husk	21.80	−2.65	7925	−24.9%
Palm fronds	21.85	−2.60	7950	−24.6%

**Figure 9:**
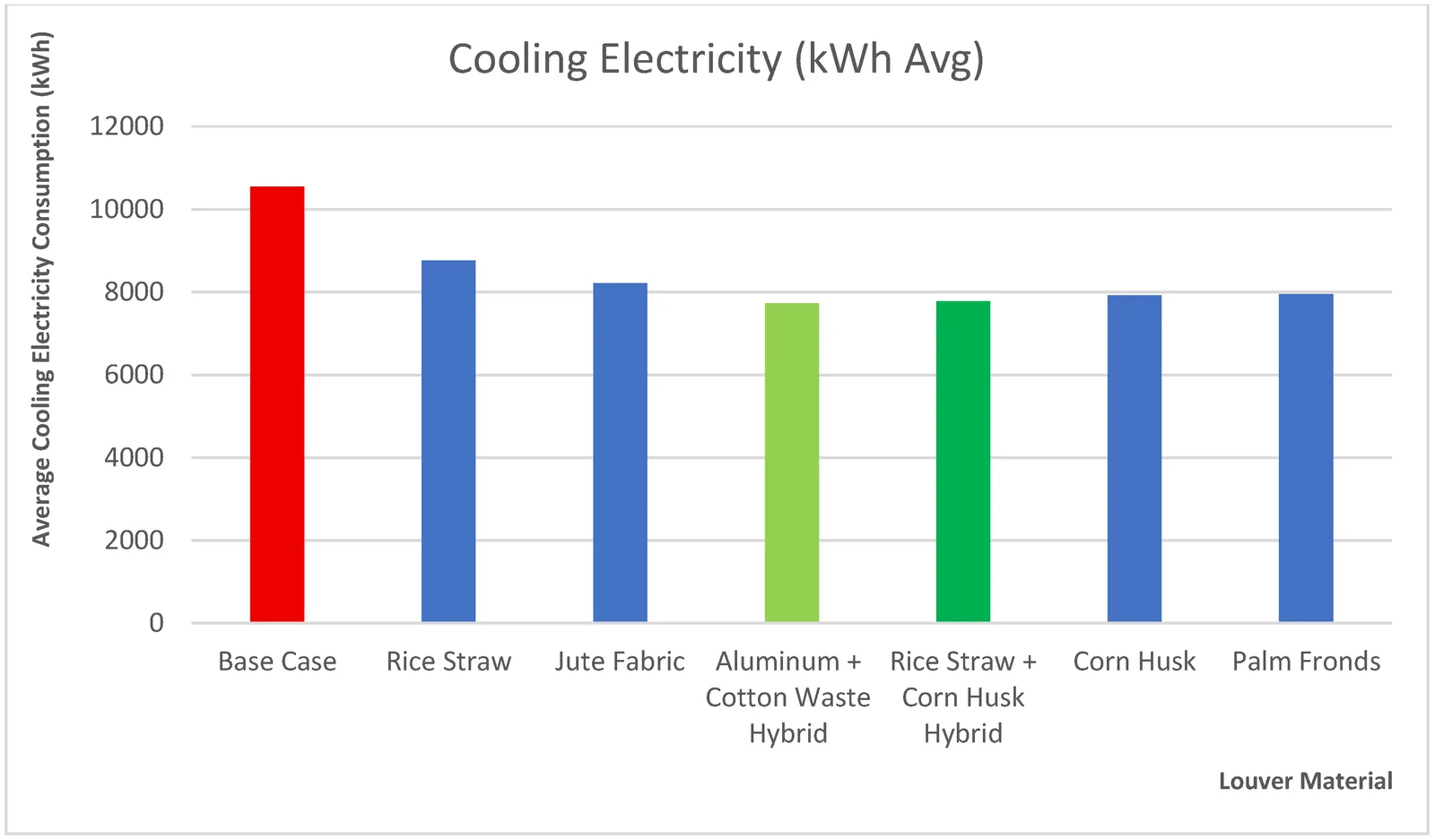
Comparison of average cooling electricity consumption for the base case and the investigated bio-inspired louver materials.

**Figure 10:**
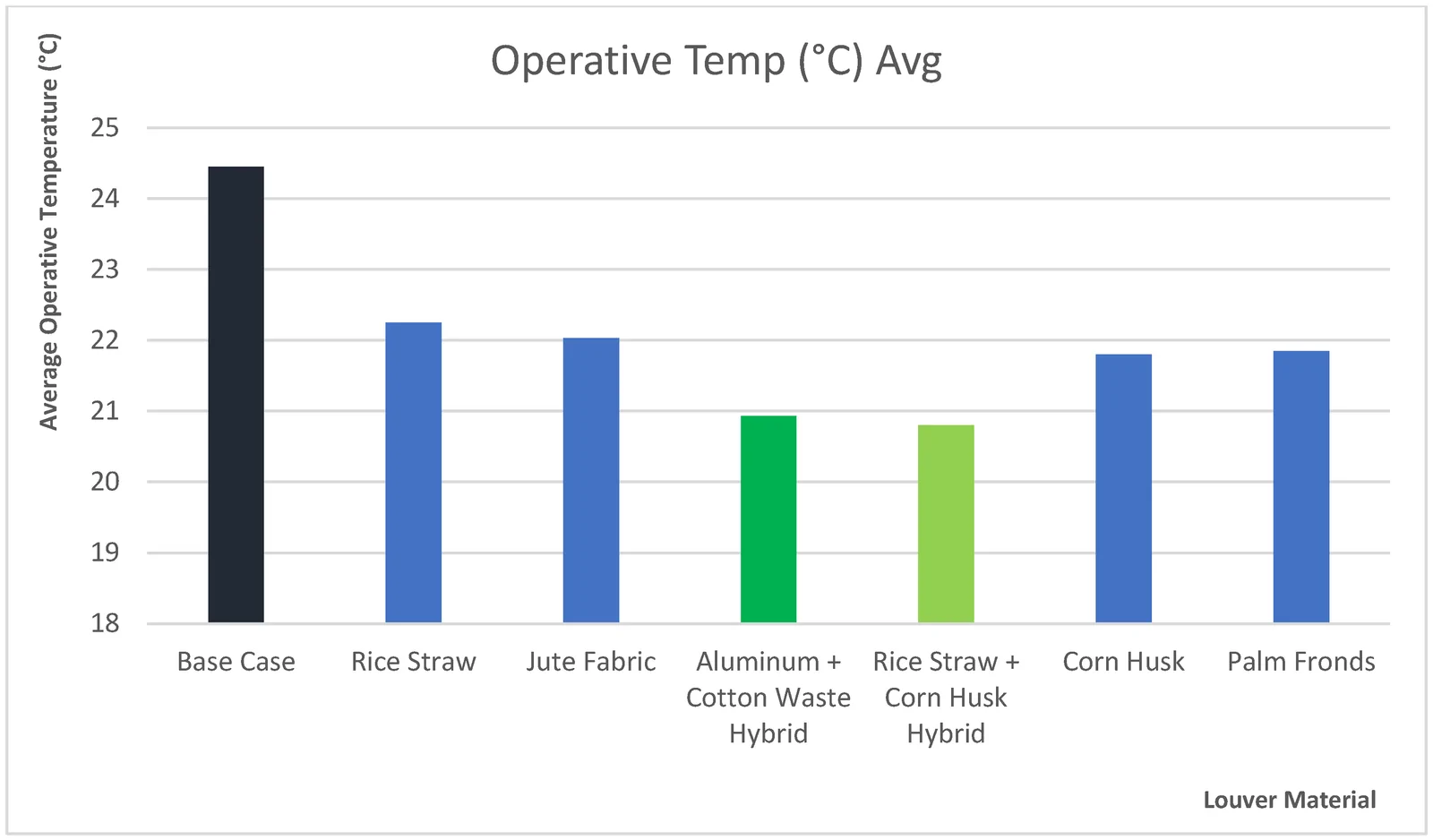
Comparison of the average operative temperature for the base case and the investigated bio-inspired louver materials.

## 6. Discussion

The simulation results indicated that the as-built residential building (base case) consumed a total of 42.2 MWh of cooling electricity in the summer period (June–September).

The biomimetic louver systems were introduced to greatly improve the energy efficiency of the building by reducing solar heat gains and cooling load. In the alternatives studied, rice straw louvers achieved a cooling electricity consumption of 35.05 MWh, thereby reducing it by around 16.9% compared to the base case. Jute-fabric louvers resulted in a further drop, the consumption being 32.85 MWh (22.2% reduction). 

The hybrid biomimetic solutions demonstrated the most potential for energy conservation, and the hybrid aluminum and cotton waste louvers achieved a significant reduction in electricity consumption for cooling to 30.9 MWh, which corresponds to nearly 26.8% energy savings compared to the baseline case. Similarly, the hybrid louvers made of rice straw and corn husk reduced the energy consumption to 31.1 MWh, a decrease of about 26.3%; the corn husk louvers reduced the consumption to 31.7 MWh (24.9% decrease), and the palm frond louvers achieved significant thermal enhancement, with cooling electricity consumption decreasing to about 31.8 MWh, a saving of about 24.6% compared to the baseline scenario. 

These findings are in accordance with prior research that has illustrated the efficacy of bio-based insulation materials and external shading devices in reducing cooling loads in hot-arid climates. Previous research has demonstrated that the cooling energy demand of Egyptian residential buildings can be significantly reduced by enhancing the building envelope [88,89,90]. Similar to this, research has indicated that the porous microstructure of natural-fiber insulation materials can improve thermal resistance [53]. The thermal advantages of rice-straw insulation materials in humid regions have been emphasized in other research [53]. The current research is based on these discoveries and combines biomimetic design principles with agricultural waste materials that are sourced from the region to create an external louver retrofit system.

In addition to energy savings, all biomimetic louver systems improved indoor thermal comfort by decreasing the average operative temperature by approximately 2.2–3.65 °C in comparison to the baseline scenario. 

The suggested retrofitting strategy would finally contribute to wider sustainability targets through the reduction of operational energy consumption and greenhouse gas emissions, as well as the extension of the life span of existing residential buildings. 

The results indicate that the choice of shading materials is of crucial importance for the energy performance of buildings. Suitable biomimetic louver materials can significantly reduce solar heat gains, cooling demands, electricity use, and associated CO_2_ emissions in hot-arid climates, thereby enhancing the energy efficiency of residential buildings. 

Future work could look at the impacts of different louver configurations, including perforated blades, different blade angles, and sliding louver configurations, as well as adaptive control strategies based on the thermophysical properties of bio-based materials such as density, thermal conductivity, solar reflectance, and air permeability. These questions could result in better thermal and energy performance of biomimetic façade systems in different climates.

The practical application of the proposed louver retrofit system is also contingent upon climatic resistance and material durability. The long-term performance of the materials under investigation may be influenced by high solar exposure, elevated temperatures, dust accumulation, and periodic humidity fluctuations, as they are designed for external use in the hot-arid climate of New Cairo. Consequently, the bio-based materials that were chosen were assessed not only in terms of their thermal efficacy but also in terms of their compatibility with the local environmental conditions. Nevertheless, the current investigation is primarily simulation-based and does not incorporate experimental long-term weathering or durability testing. Consequently, it is advised that additional experimental research be conducted to assess the mechanical durability, moisture resistance, UV stability, and aging of the proposed materials in real-world outdoor environments.

## 7. Conclusions

This research demonstrates the benefits of implementing passive design in buildings over conventional construction materials, as this strategy will diminish energy consumption, subsequently reducing greenhouse gas emissions in the surrounding environment and alleviating financial burdens on building owners and occupants. 

This study demonstrated the potential of biomimetic louver retrofit systems developed from locally available agricultural waste materials to improve the thermal and energy performance of residential buildings in hot-arid climates. All investigated materials reduced cooling electricity consumption and operative temperature compared with the baseline case, while the hybrid aluminum–cotton waste and rice straw–corn husk systems achieved the best overall performance. The proposed retrofit strategy provides a practical and sustainable approach for improving existing residential buildings in New Cairo and similar climatic regions.

In the present study, numerical simulation under the fixed louver geometry was carried out to clarify the influence of material properties. Hence, the optimization of louver geometry, fabrication of the prototype, and experimental validation were beyond the scope of the present research. These elements are significant avenues for future research to improve and extend the conclusions presented in this study. 

This study specifically aimed to evaluate biomimetic louver systems developed from locally available agricultural residues. Traditional shading mechanisms are effective in reducing solar heat absorption and improving the thermal efficiency of buildings, but they do not solve the environmental problem of the large quantities of agricultural waste that are annually burned in rural Egypt. The proposed method represents a step forward from thermal efficiency by converting such neglected waste materials into functional building envelope components, thus improving building energy performance, promoting circular economy practices, and contributing to the reduction of air pollution associated with open-field burning of agricultural residues. 

The results indicate that biomimetic louver retrofits using local agricultural waste materials can be an effective passive approach to enhance the thermal resilience and energy efficiency of existing residential buildings in hot-arid conditions.

## Figures and Tables

**Figure 2:**
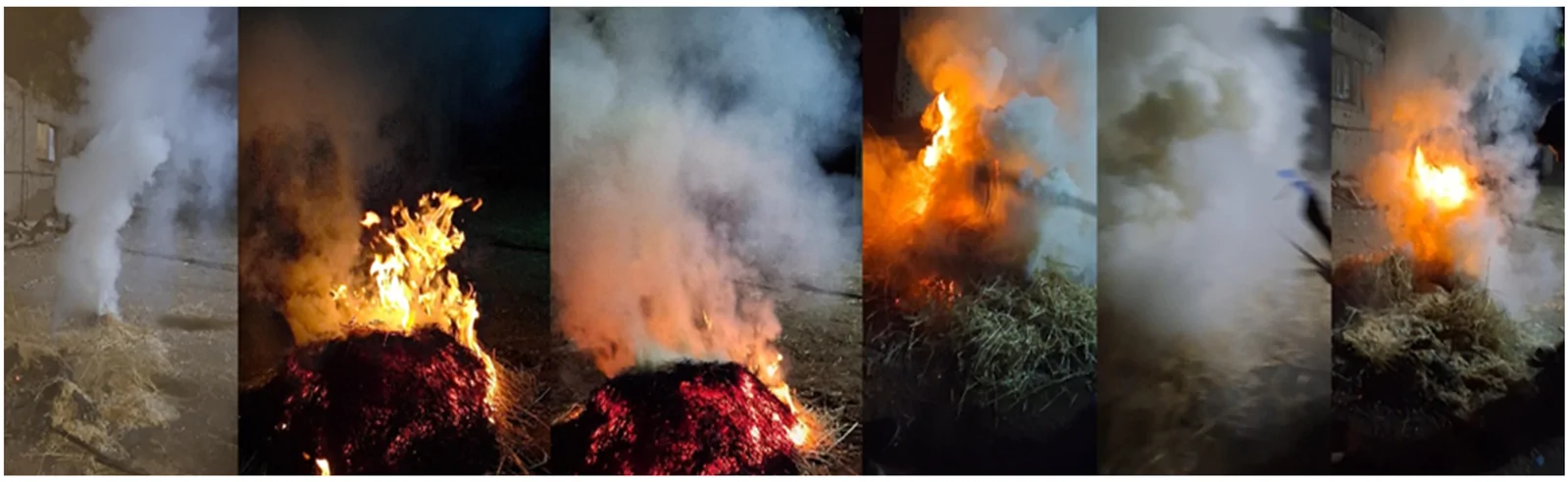
(site visit) In the open-field burning of rice straw and corn husk, the amount of smoke and CO_2_ emissions are high, and they contribute to serious air pollution, greenhouse gas accumulation, and poor health effects.

**Figure 3:**
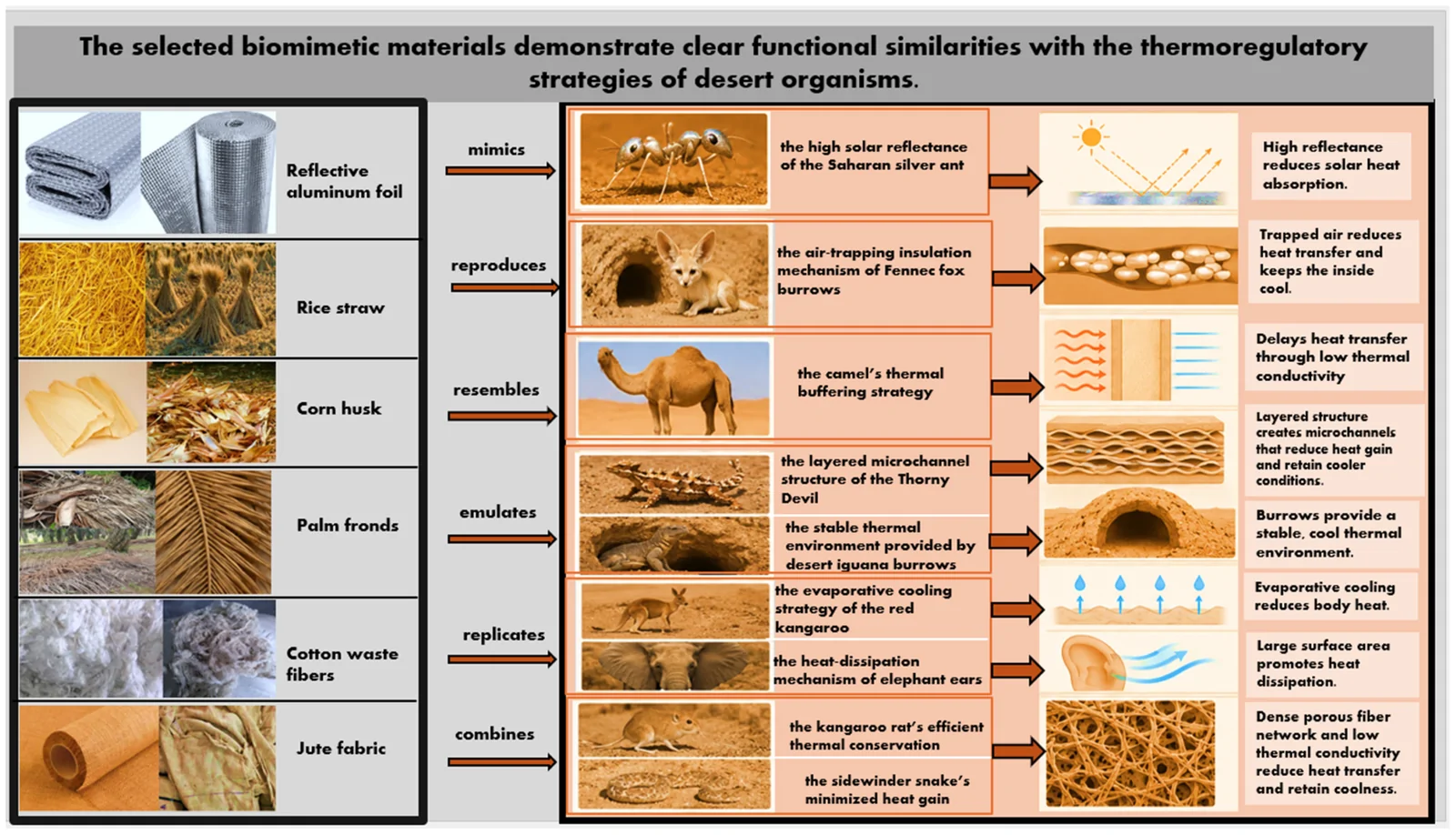
Biomimetic mapping of selected building materials to desert organisms, showing the translation of biological thermoregulation strategies into material-based passive cooling and thermal insulation mechanisms for buildings.

**Figure 4:**
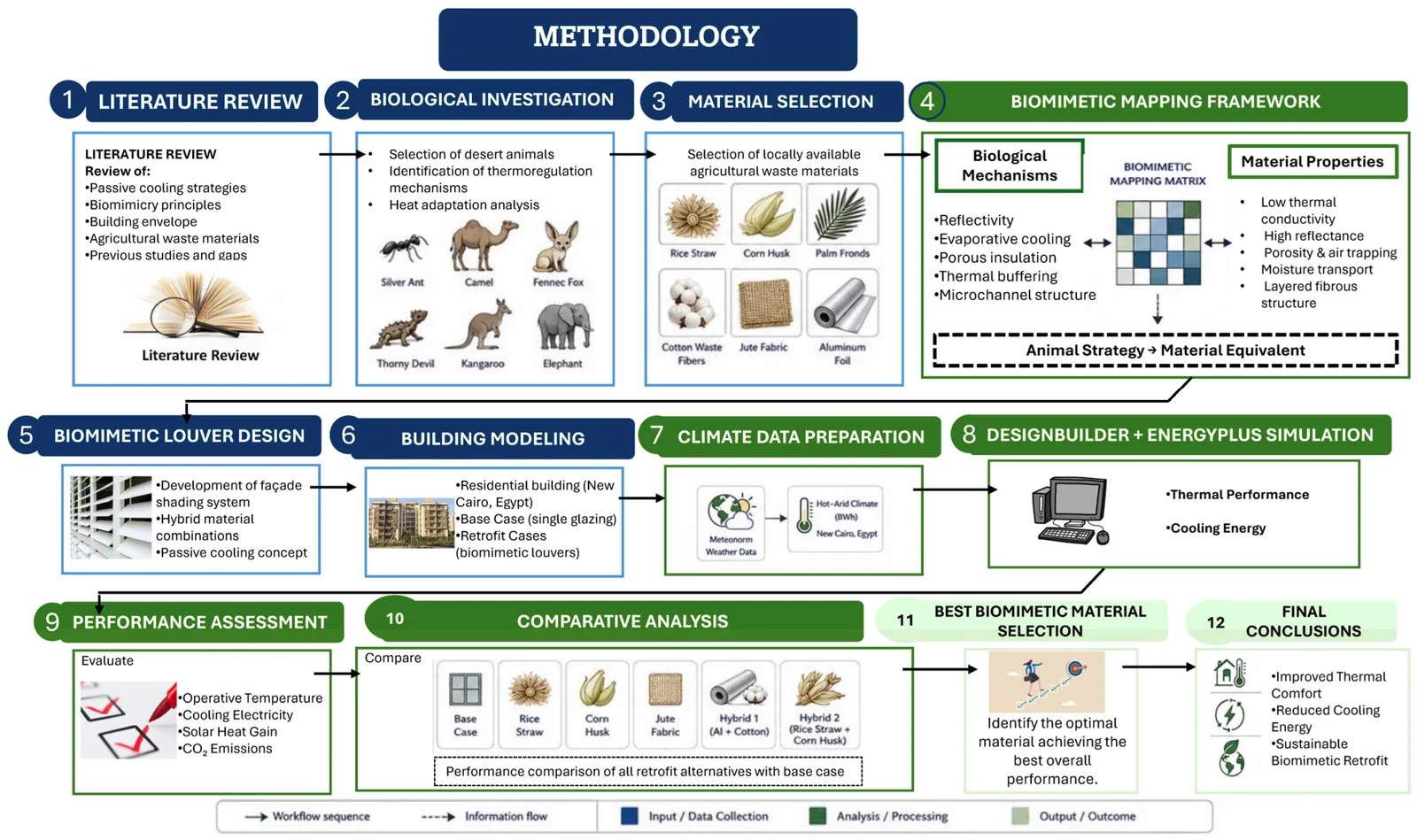
Workflow of the research.

**Figure 5:**
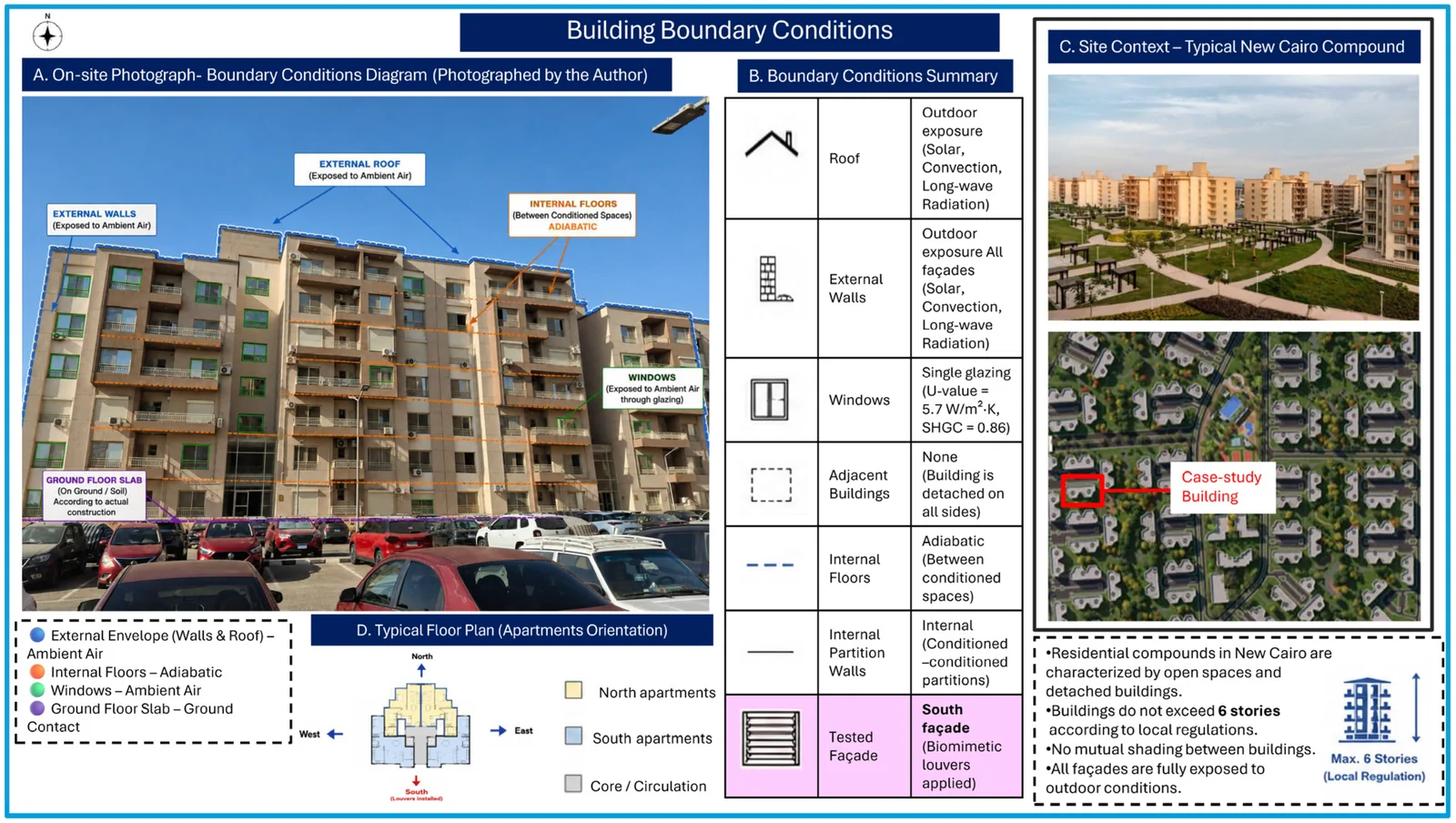
Building boundary conditions.

**Figure 7:**
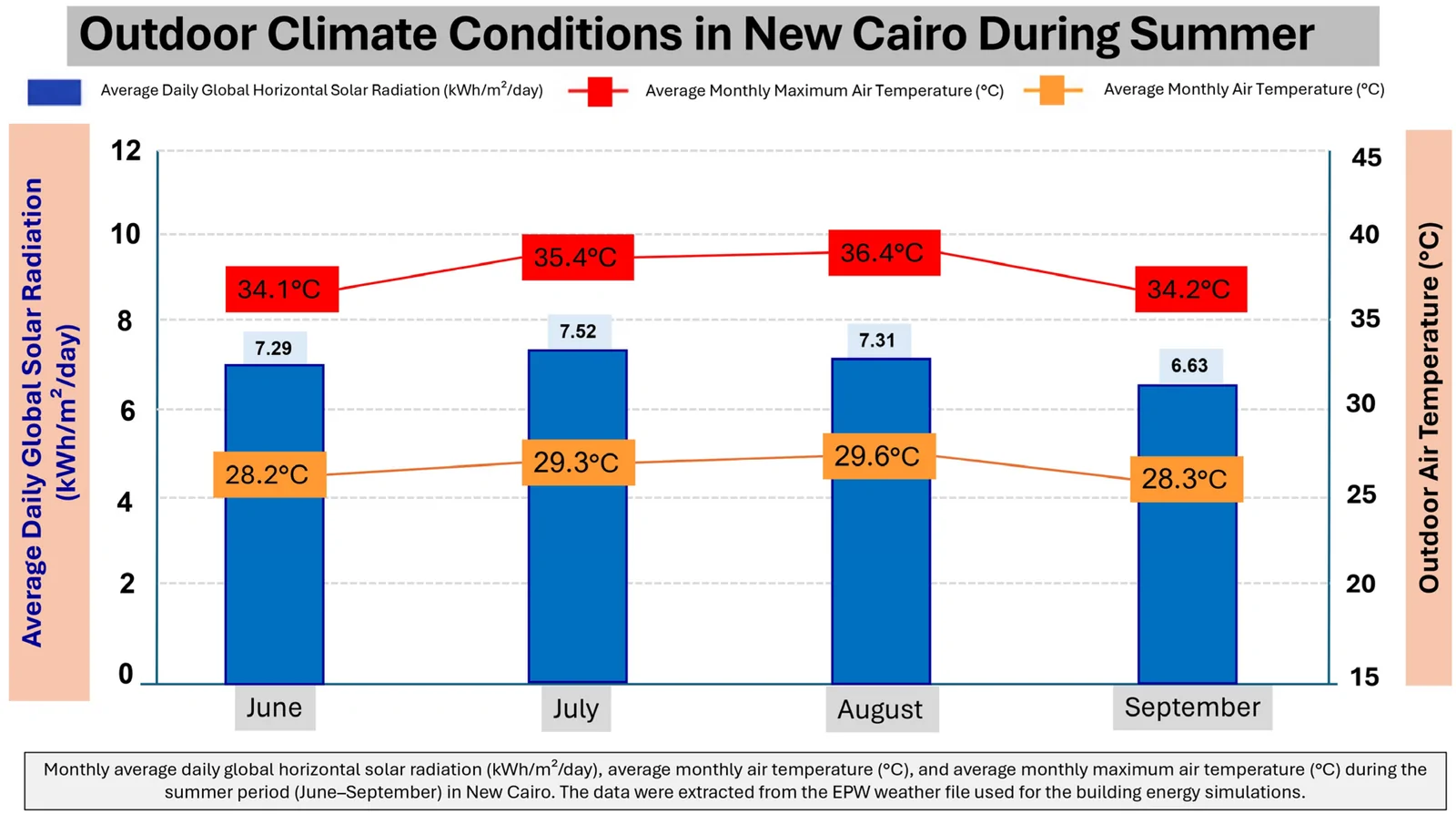
Monthly average and maximum outdoor air temperatures (°C) together with global horizontal solar radiation (kWh/m^2^/day) during the summer period (June–September) in New Cairo, Egypt. The climatic data were obtained from the Meteonorm weather database and used as the weather input for the DesignBuilder/EnergyPlus simulations.

**Table 1:** Research Gaps in Biomimicry for Energy Performance.

Research Area	Current Status	Gap Identified
Building Type	Focused primarily on new or landmark developments	Limited application to existing residential building stock
Climate Context	Numerous studies in temperate climates	Limited studies in hot-arid desert climates
Simulation	Studies mainly investigate single biomimetic strategies.	Lack of comparative simulation of multiple biomimetic strategies
Scalability	Conceptual/experimental models	Limited guidance for large-scale retrofitting of existing residential buildings

**Figure A1:**
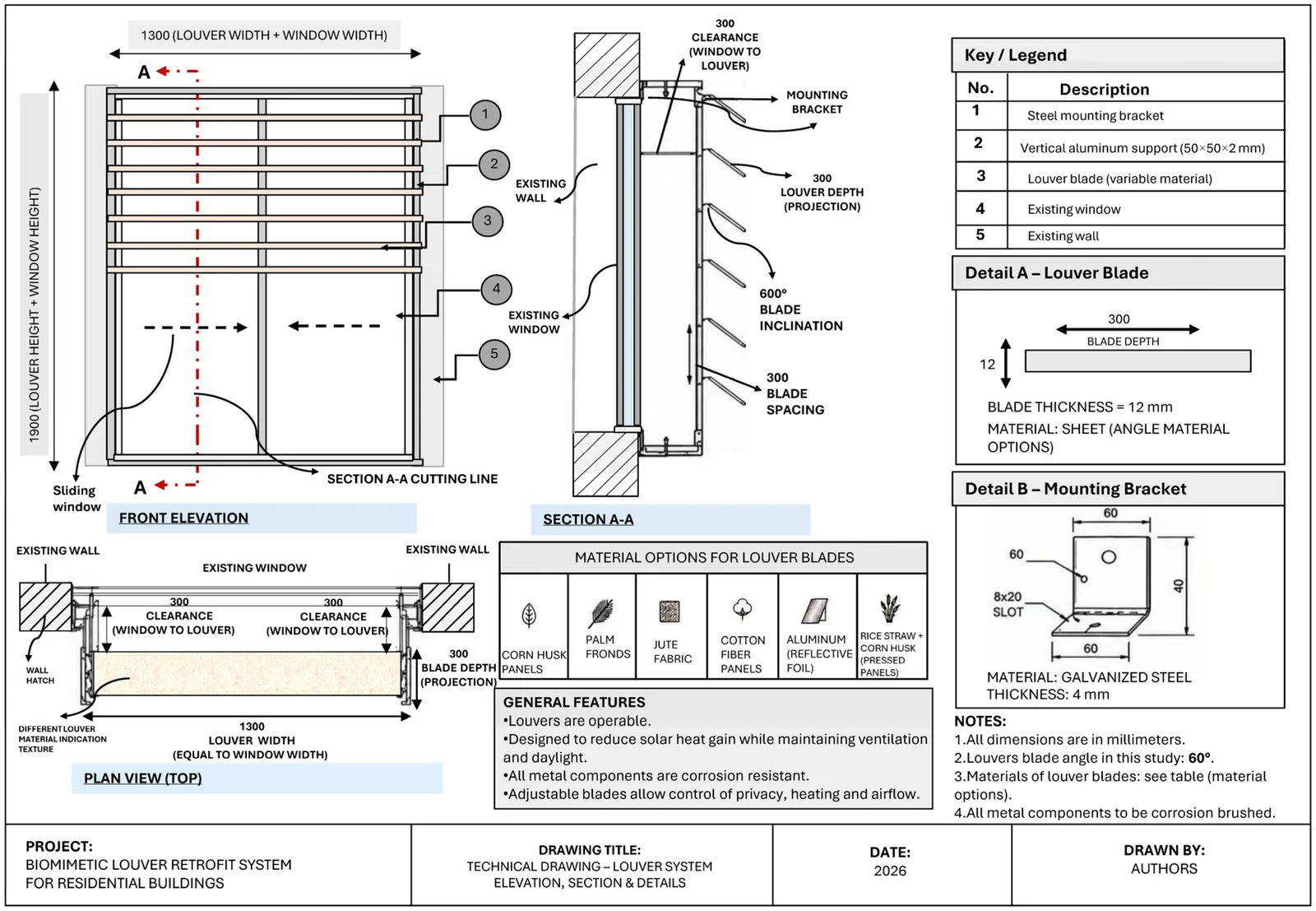
Proposed biomimetic louver retrofit conceptual installation and construction details, including mounting system details, cross-sectional geometry, window operability, and solar-shading concept. Source: Author.

**Figure A2:**
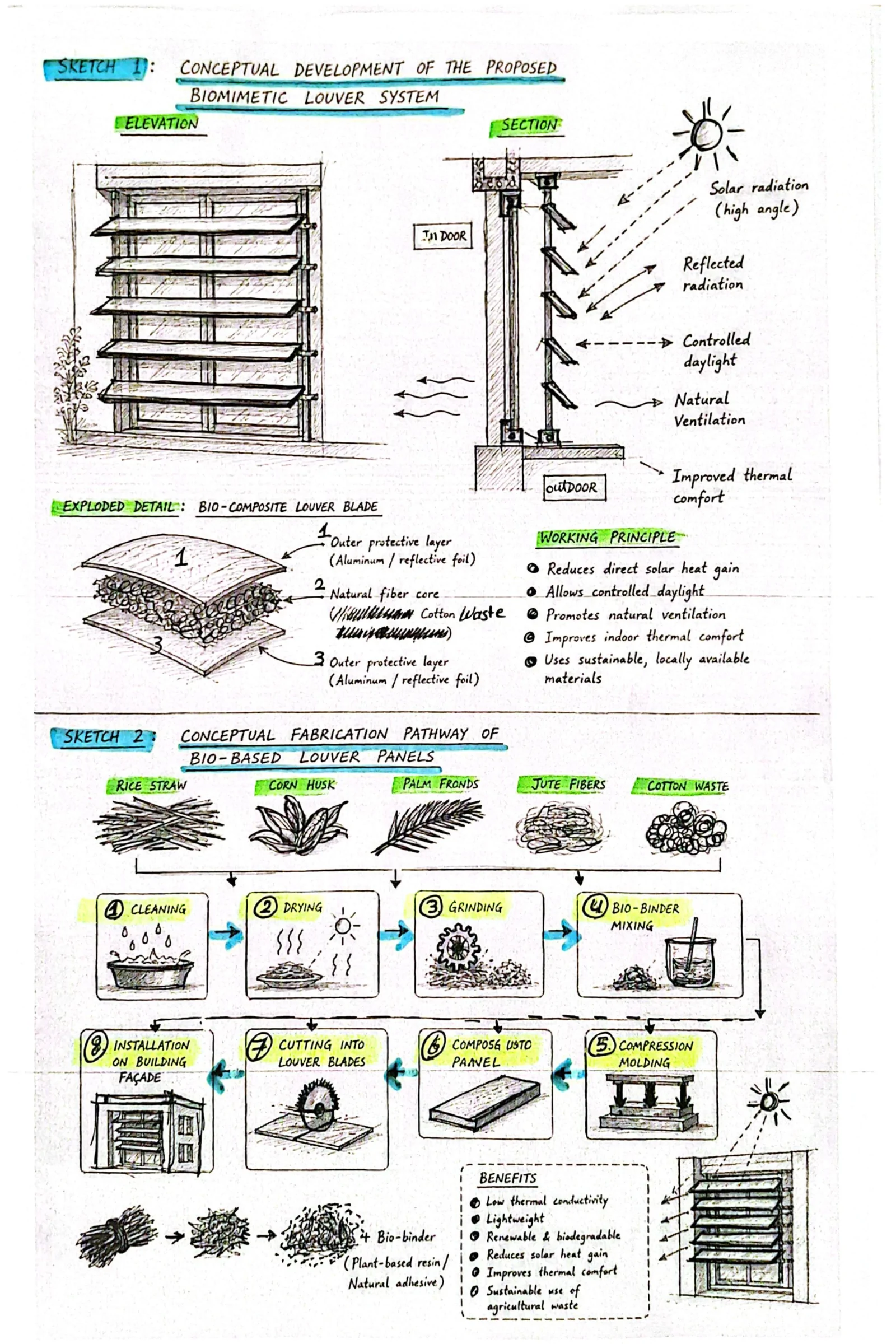
The proposed bio-based louver panel fabrication process is illustrated in a schematic diagram, which is based on conventional natural-fiber composite manufacturing methods. Source: The author’s own work.

**Figure A3:**
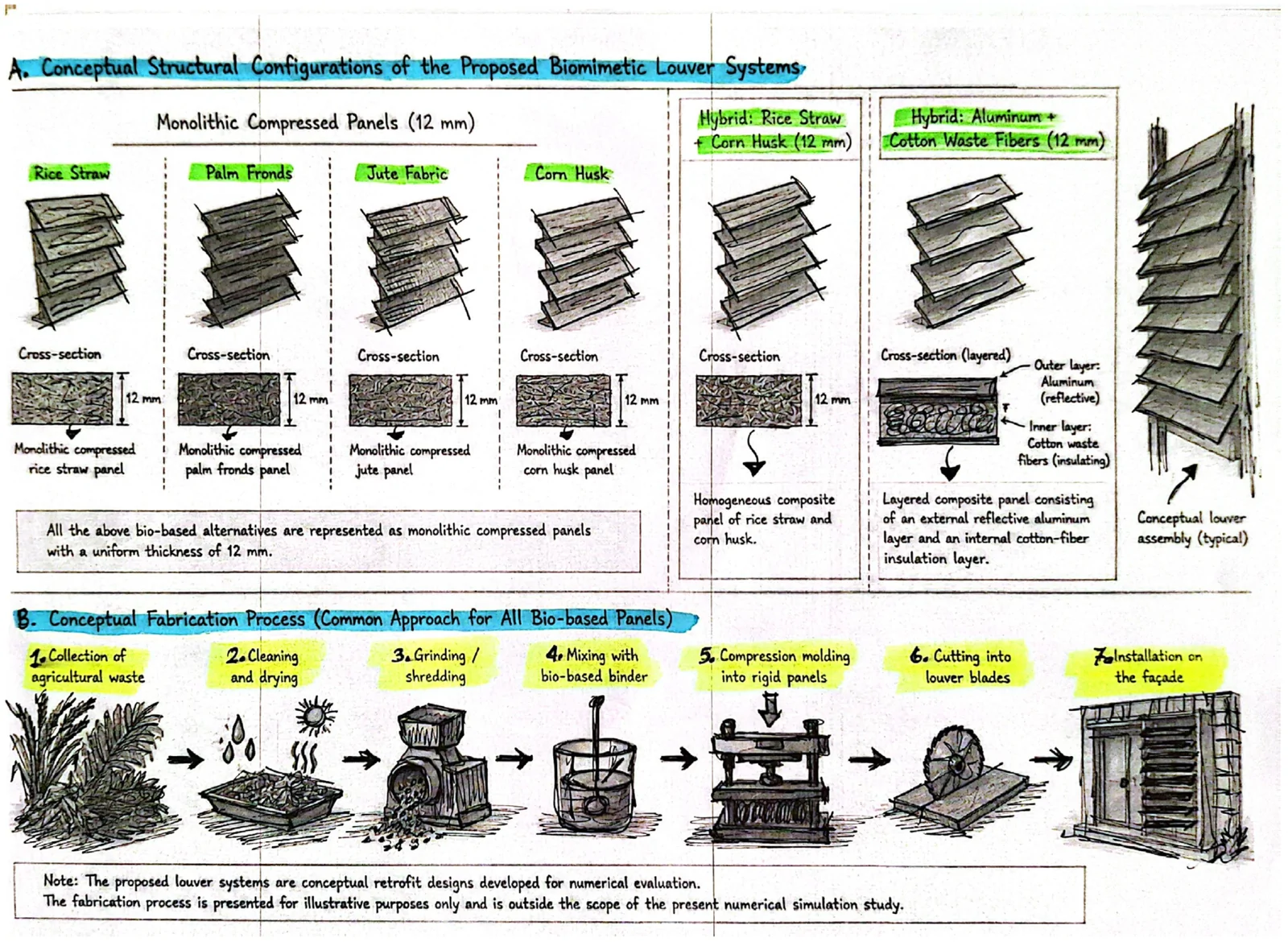
Conceptual fabrication pathway of the proposed bio-based louver panel based on conventional natural-fiber composite manufacturing techniques. Source: Prepared by the authors.

**Figure A4:**
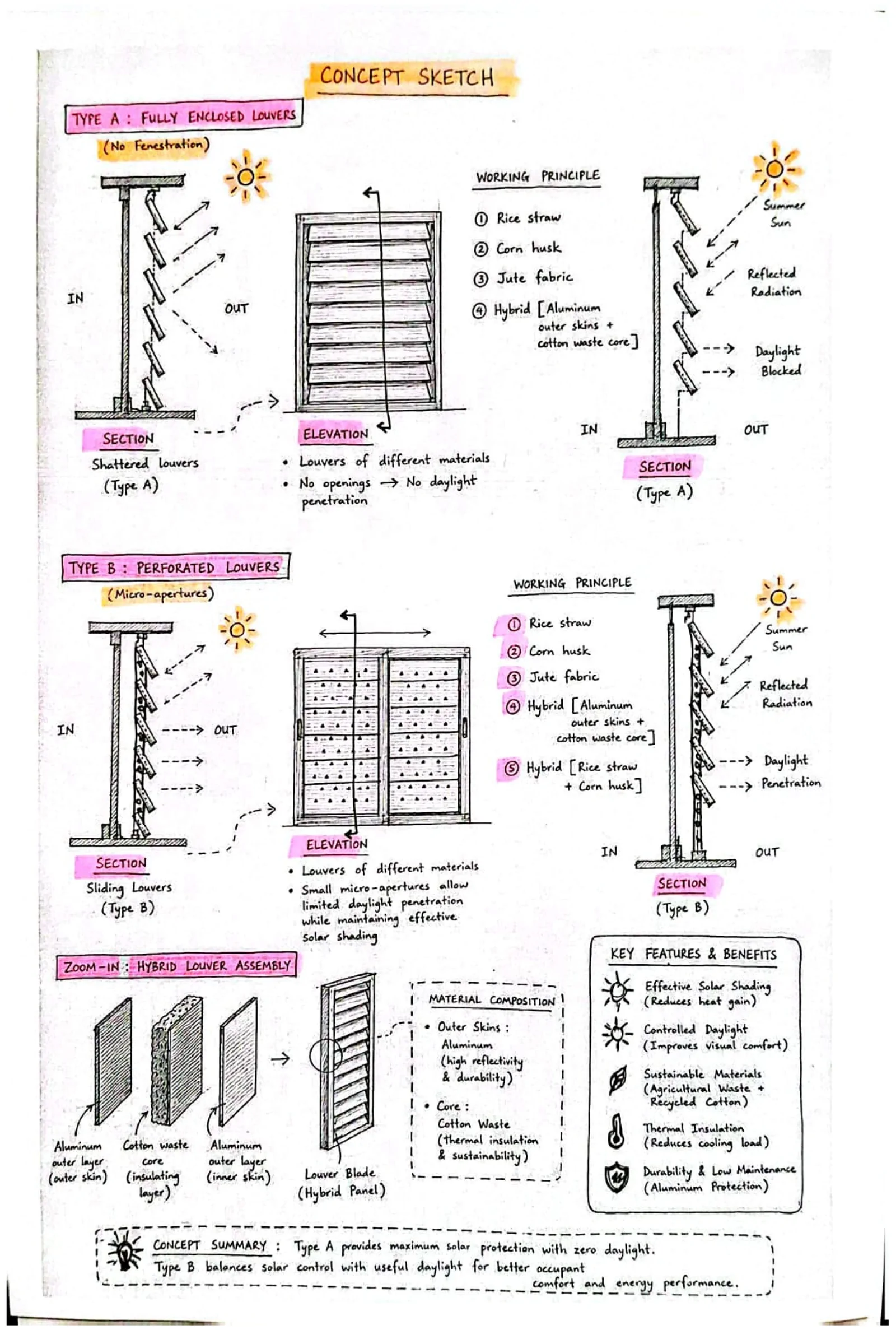
Conceptual design of the proposed biomimetic louver system. Fully enclosed and perforated louver configurations, the associated solar-shading principles, and the proposed sustainable material combinations. Source: Prepared by the authors.

**Figure A5:**
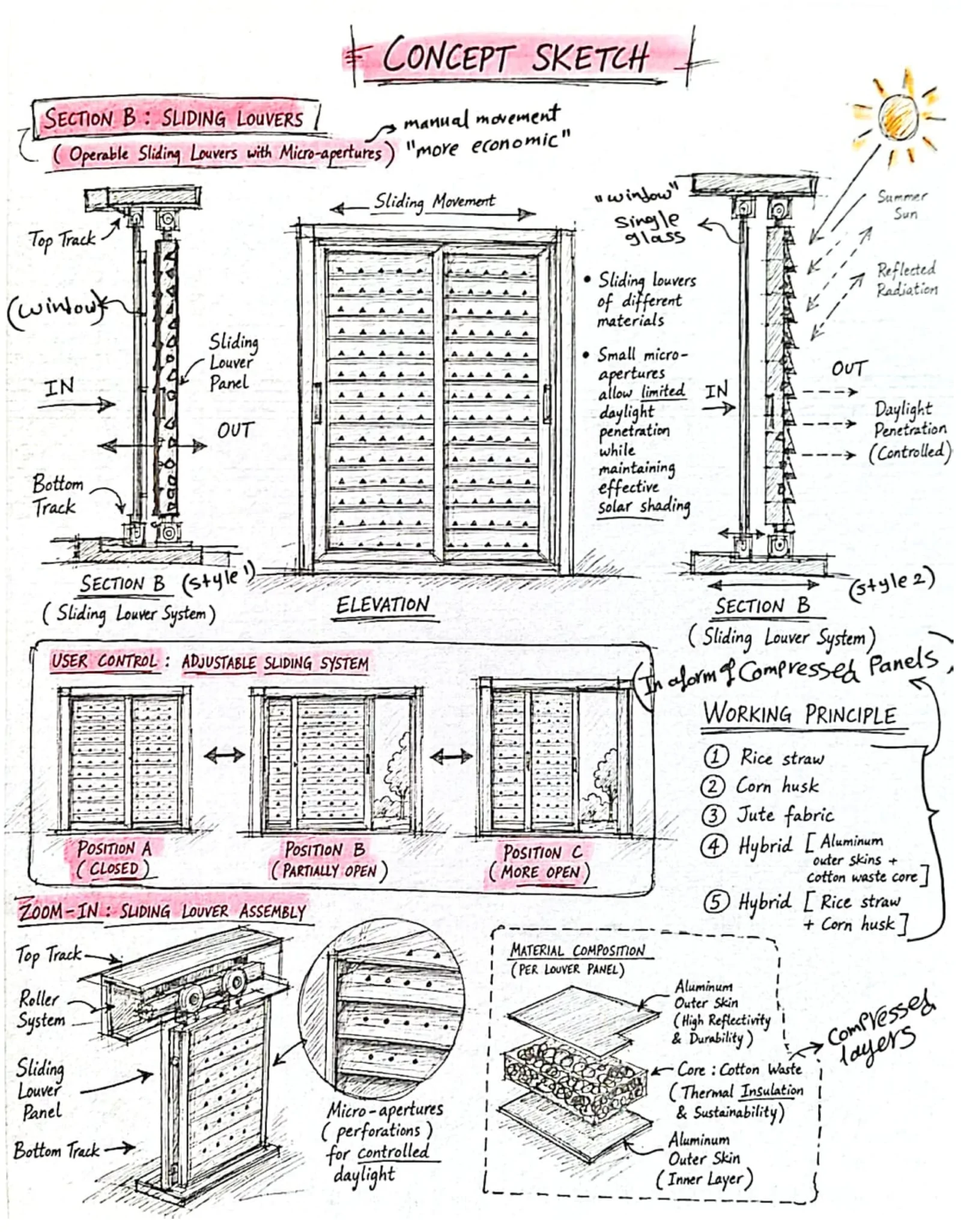
Conceptual design of the operable sliding louver system with micro-apertures Sliding mechanism, adjustable shading positions, and the hybrid louver panel assembly. Source: Prepared by the authors.

**Figure A6:**
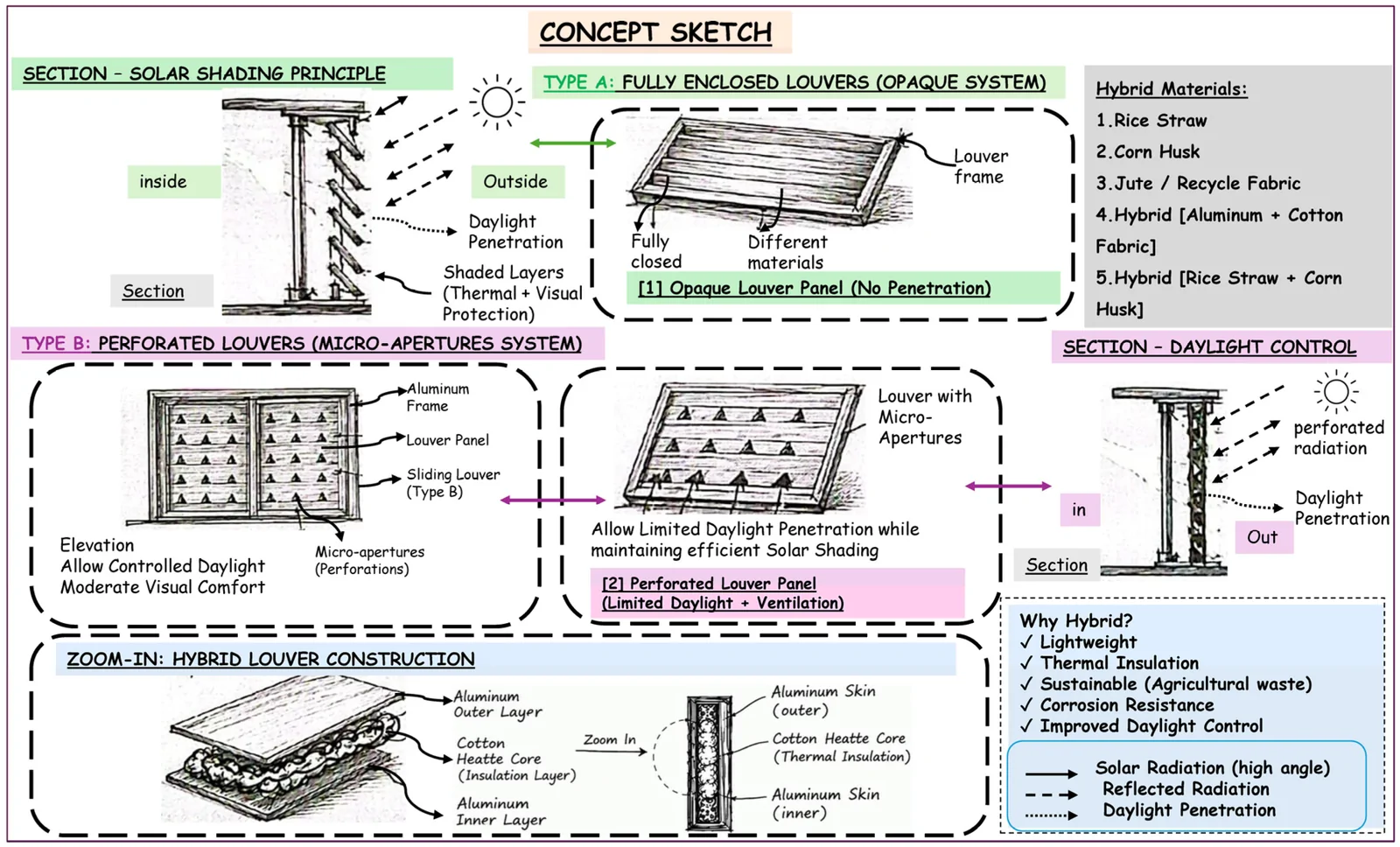
Refined concept development of the proposed biomimetic louver system, illustrating the fully enclosed and perforated configurations, solar-shading and daylight-control principles, and the hybrid louver construction. Source: Prepared by the authors.

**Figure A7:**
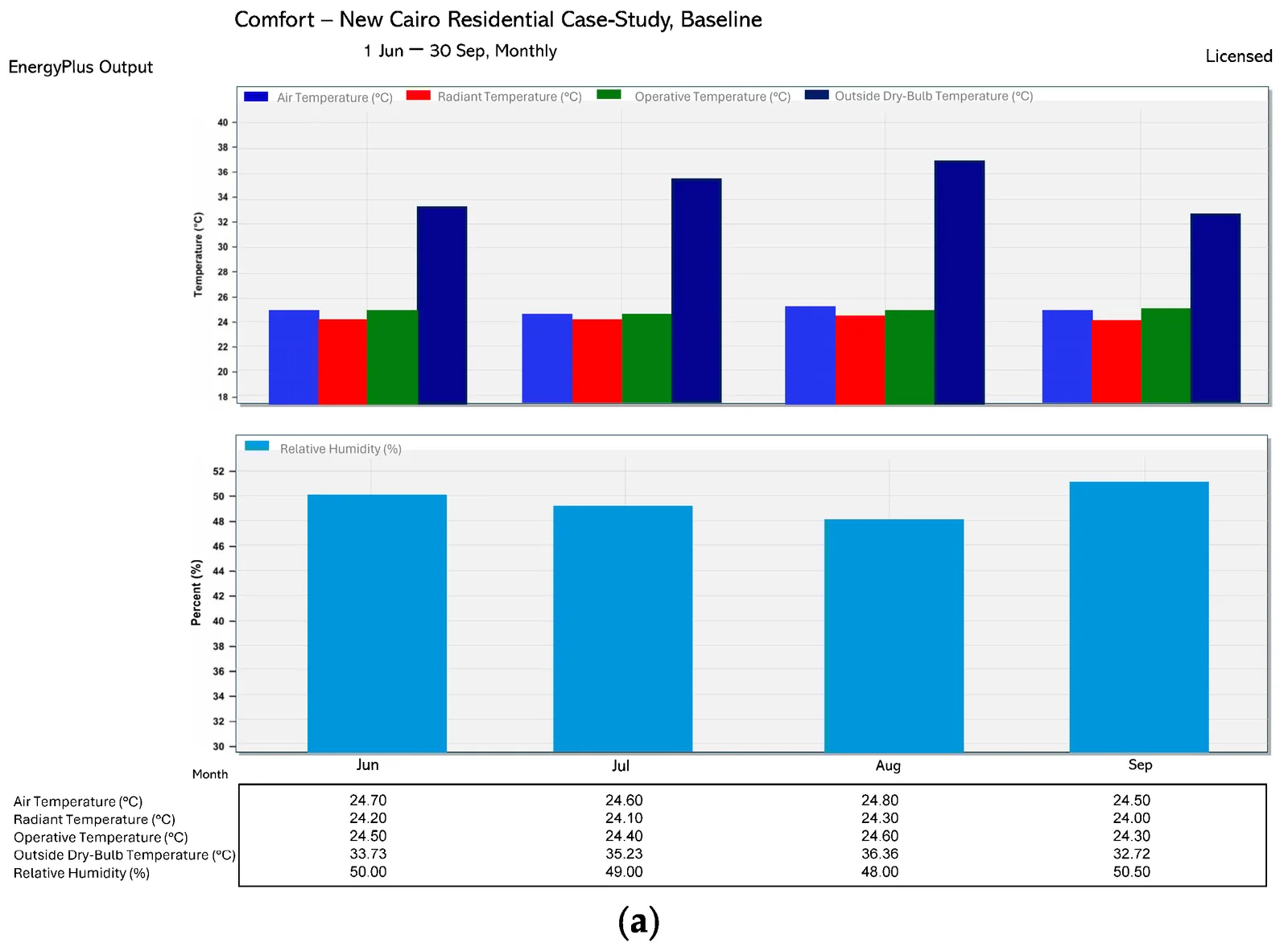

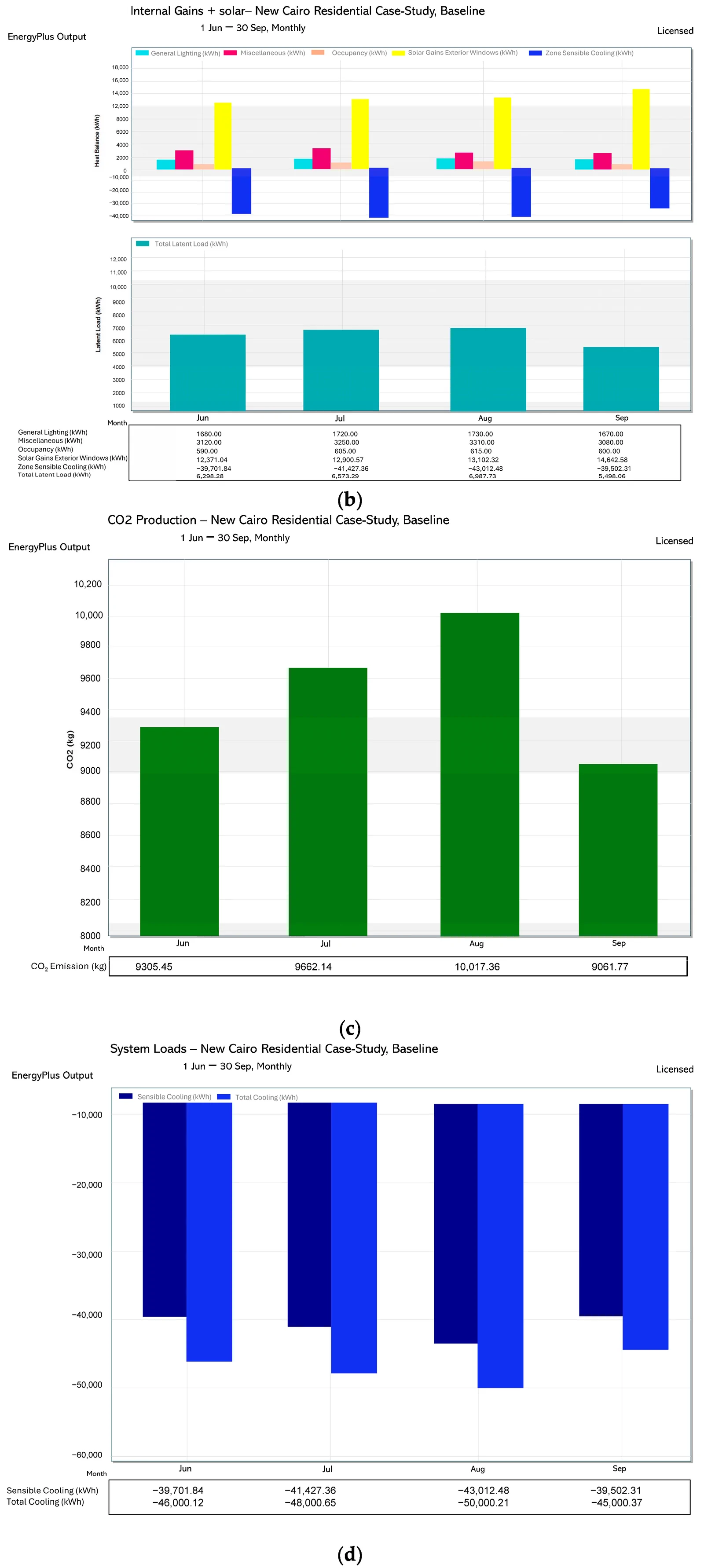

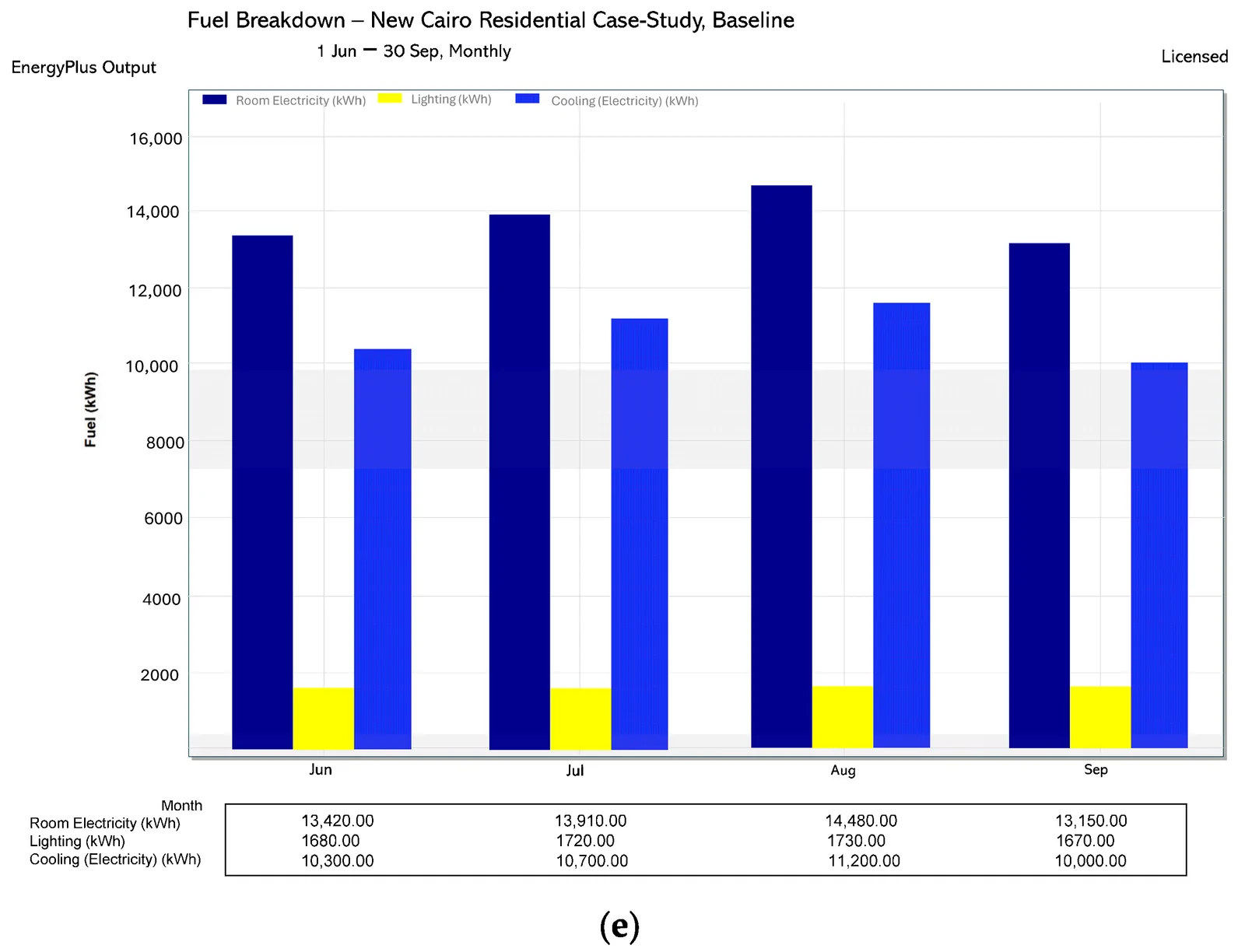
(**a**) indoor thermal comfort and temperature indicators. (**b**) internal and solar heat gains and total latent load. (**c**) operational CO_2_ emissions. (**d**) sensible and total cooling loads. (**e**) fuel breakdown, including room electricity, lighting, and cooling electricity consumption.

**Figure A8:**
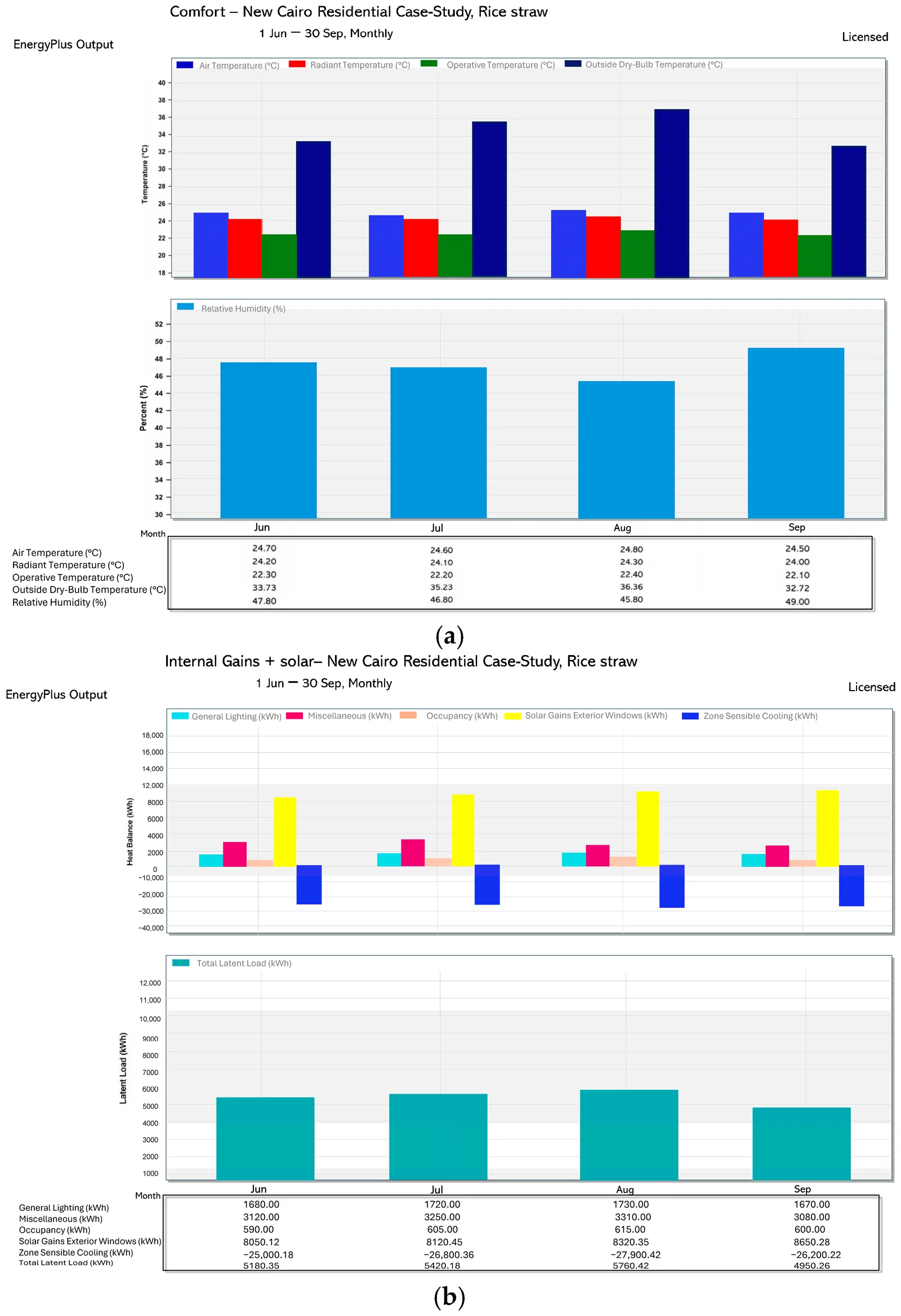

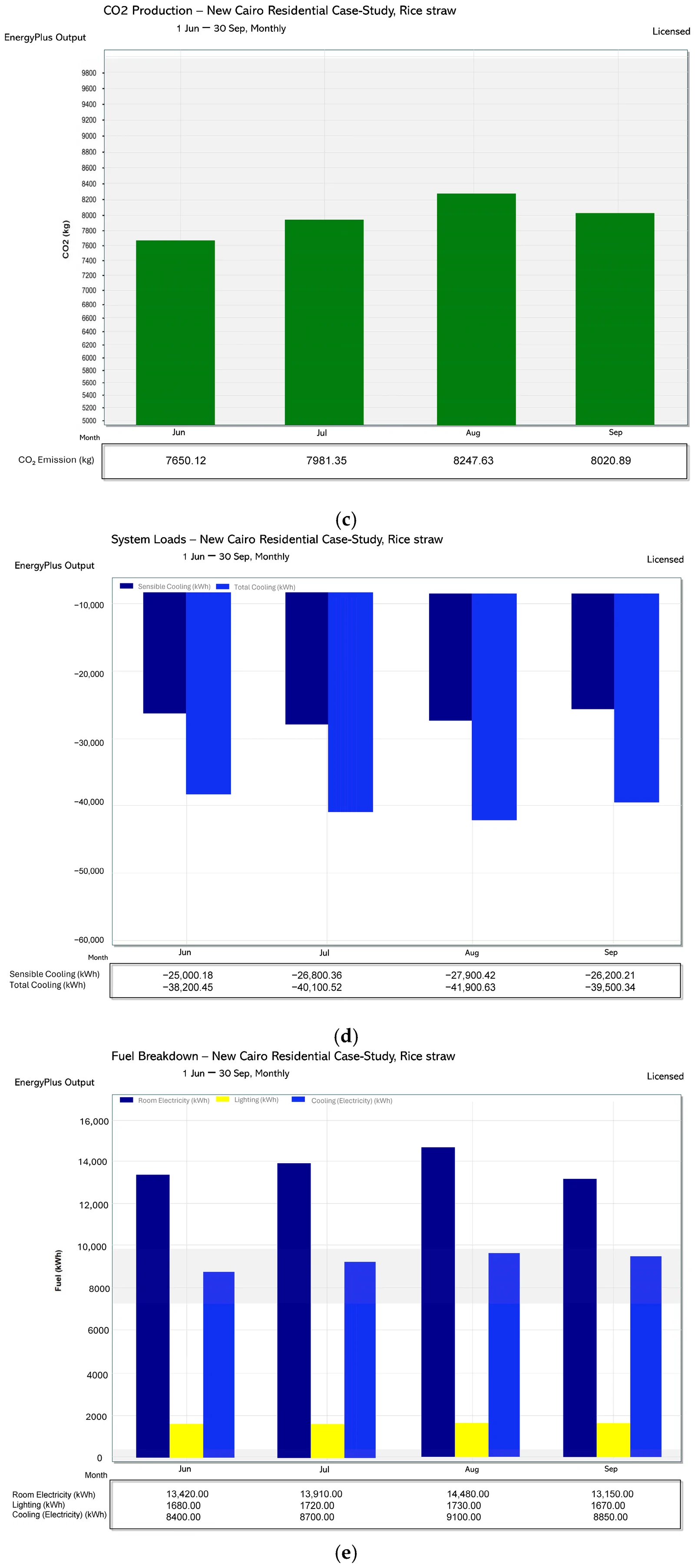
(**a**) indoor thermal comfort and temperature indicators. (**b**) internal and solar heat gains and total latent load. (**c**) operational CO_2_ emissions. (**d**) sensible and total cooling loads. (**e**) fuel breakdown, including room electricity, lighting, and cooling electricity consumption.

**Figure A9:**
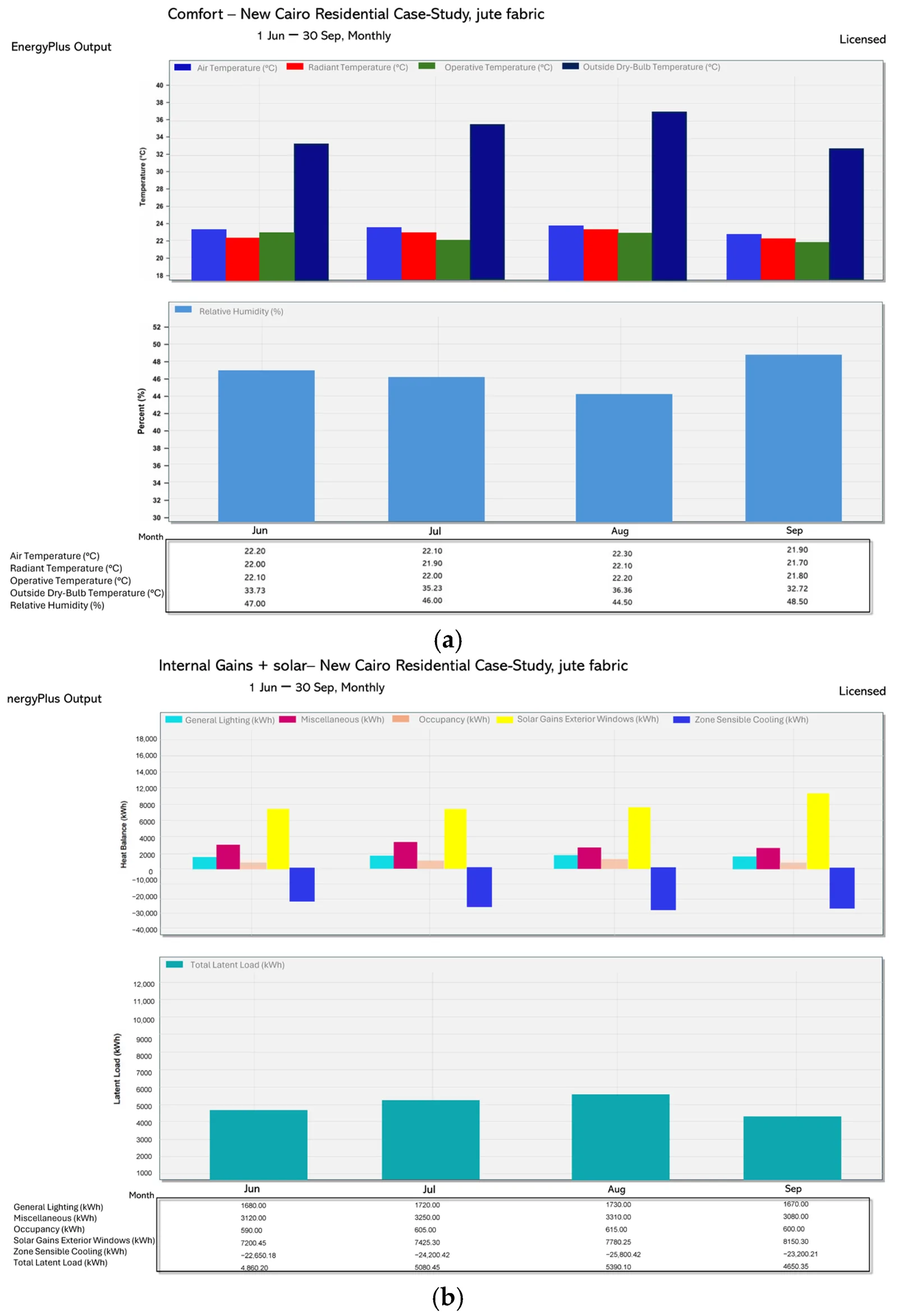

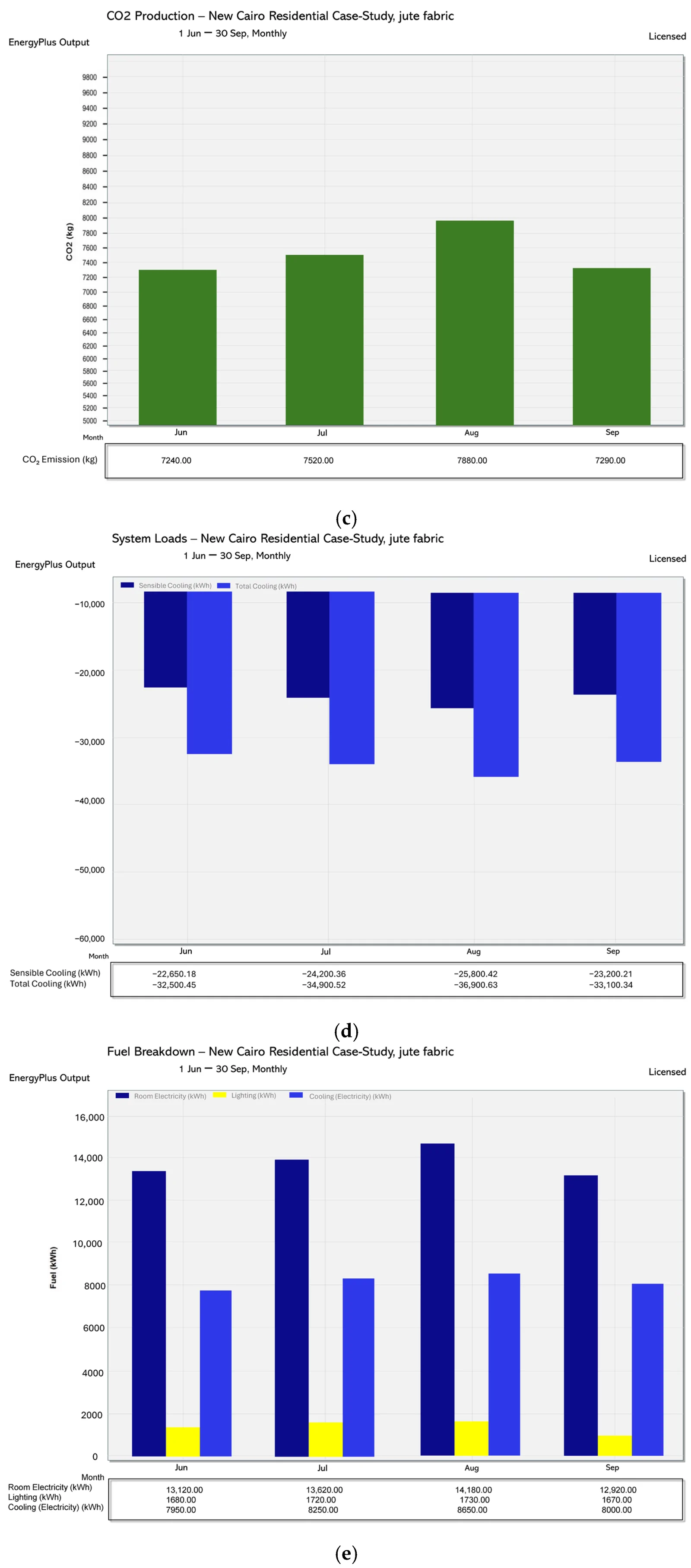
(**a**) indoor thermal comfort and temperature indicators. (**b**) internal and solar heat gains and total latent load. (**c**) operational CO_2_ emissions. (**d**) sensible and total cooling loads. (**e**) fuel breakdown, including room electricity, lighting, and cooling electricity consumption.

**Figure A10:**
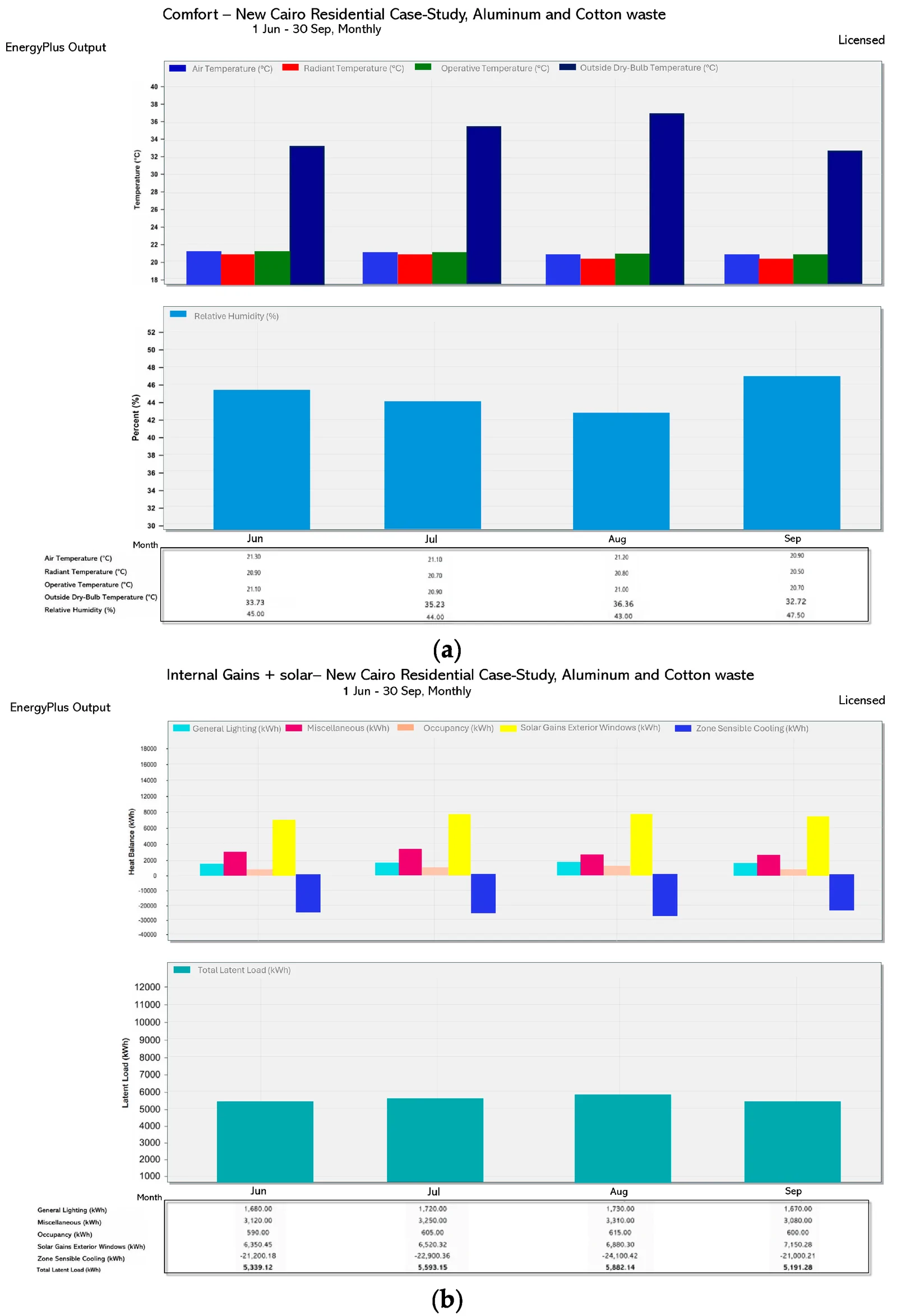

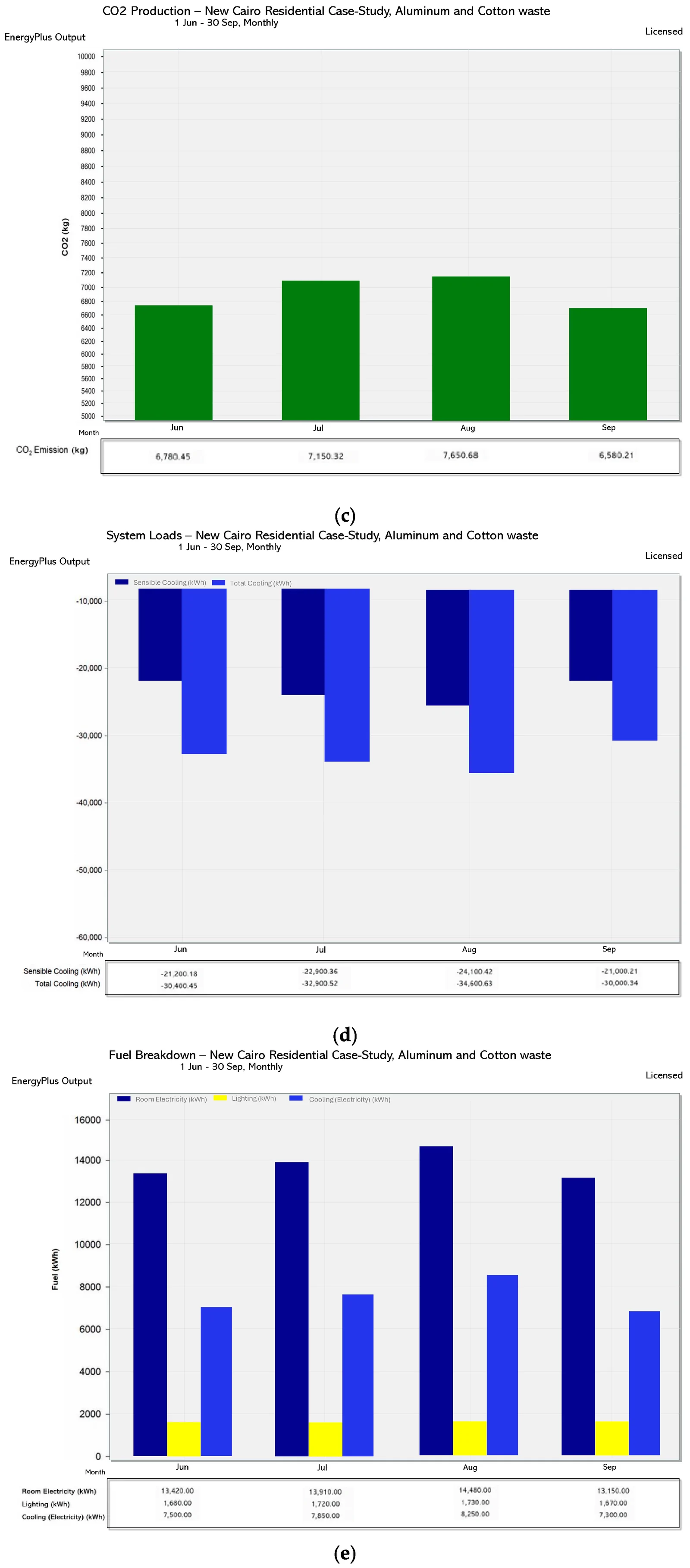
(**a**) indoor thermal comfort and temperature indicators. (**b**) internal and solar heat gains and total latent load. (**c**) operational CO_2_ emissions. (**d**) sensible and total cooling loads. (**e**) fuel breakdown, including room electricity, lighting, and cooling electricity consumption.

**Figure A11:**
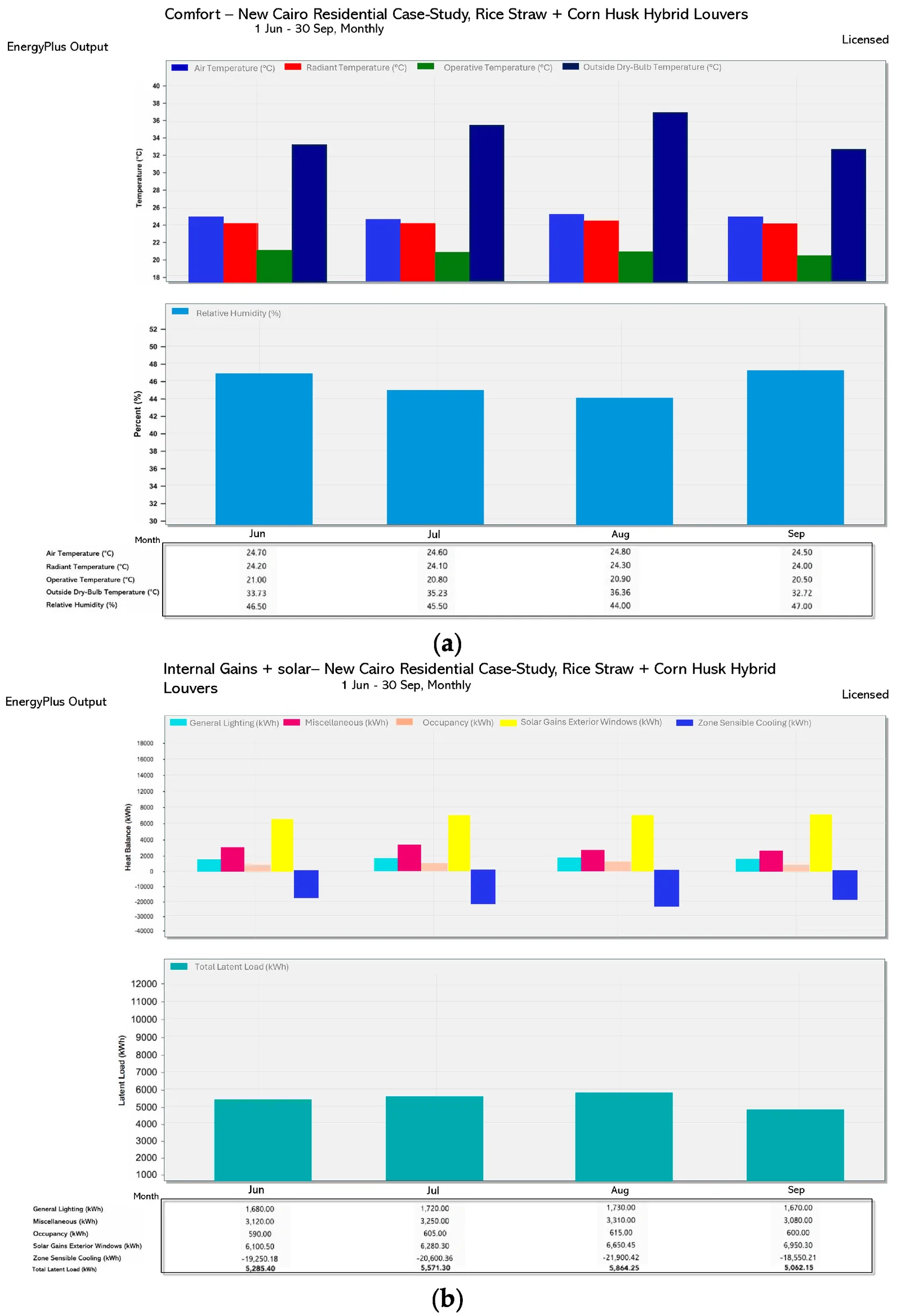

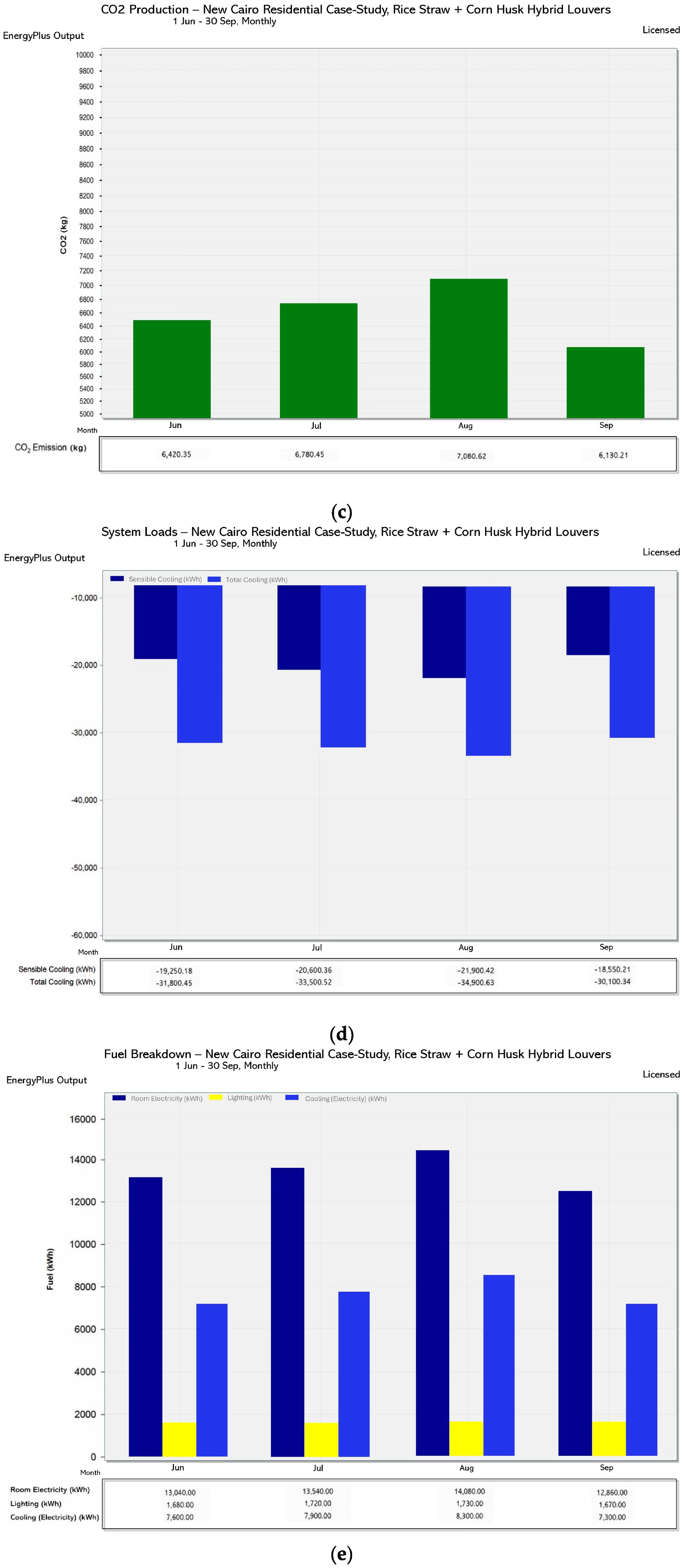
(**a**) indoor thermal comfort and temperature indicators. (**b**) internal and solar heat gains and total latent load. (**c**) operational CO_2_ emissions. (**d**) sensible and total cooling loads. (**e**) fuel breakdown, including room electricity, lighting, and cooling electricity consumption.

**Figure A12:**
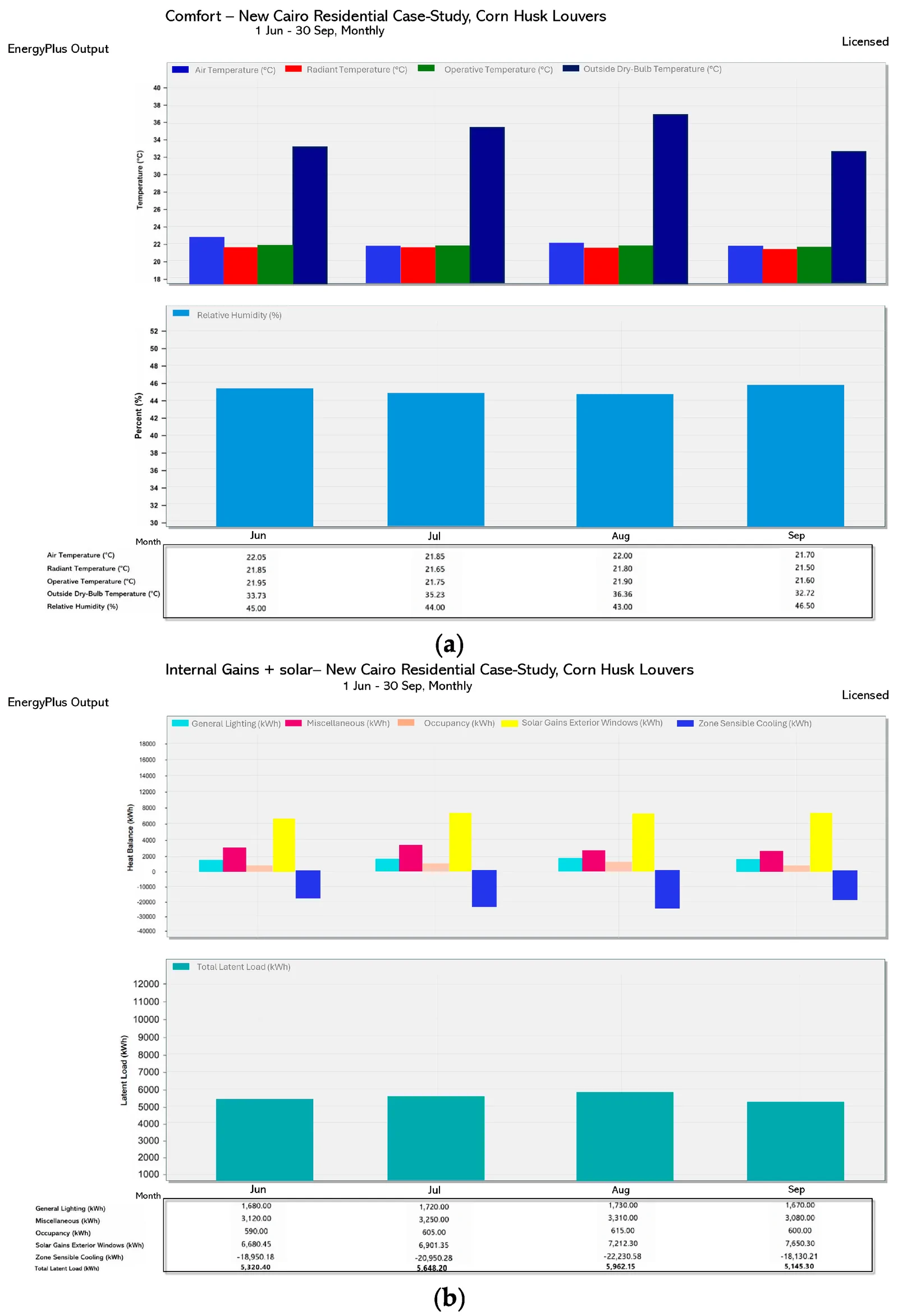

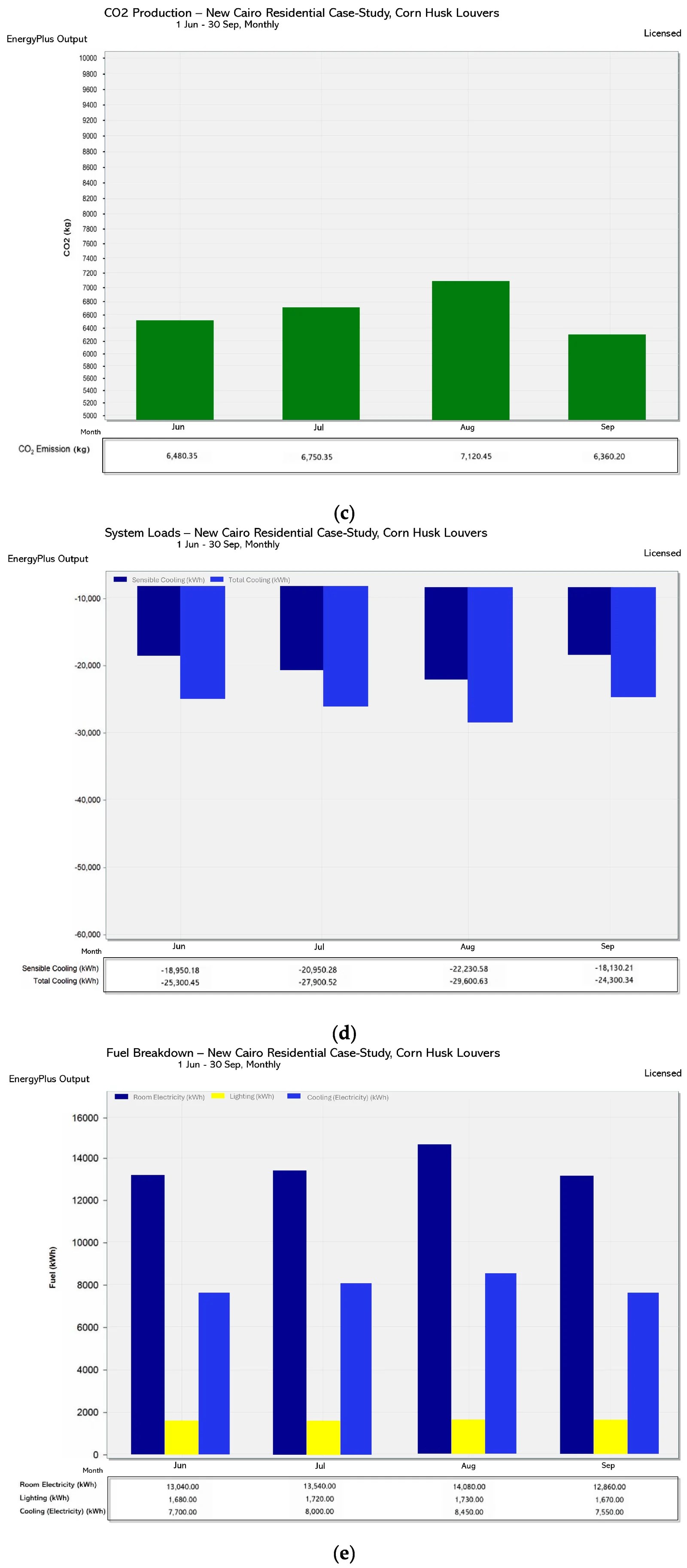
(**a**) indoor thermal comfort and temperature indicators. (**b**) internal and solar heat gains and total latent load. (**c**) operational CO_2_ emissions. (**d**) sensible and total cooling loads. (**e**) fuel breakdown, including room electricity, lighting, and cooling electricity consumption.

**Figure A13:**
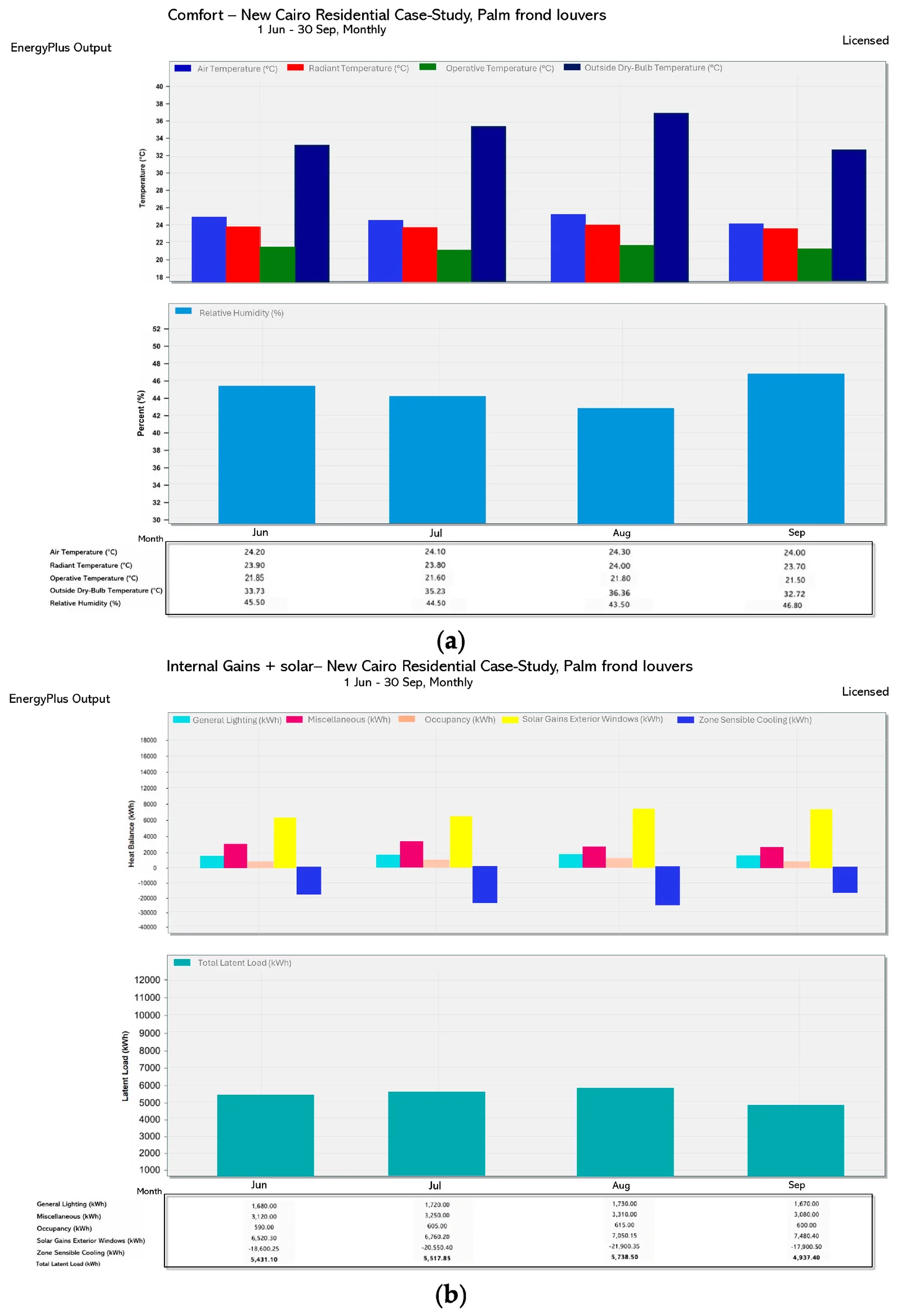

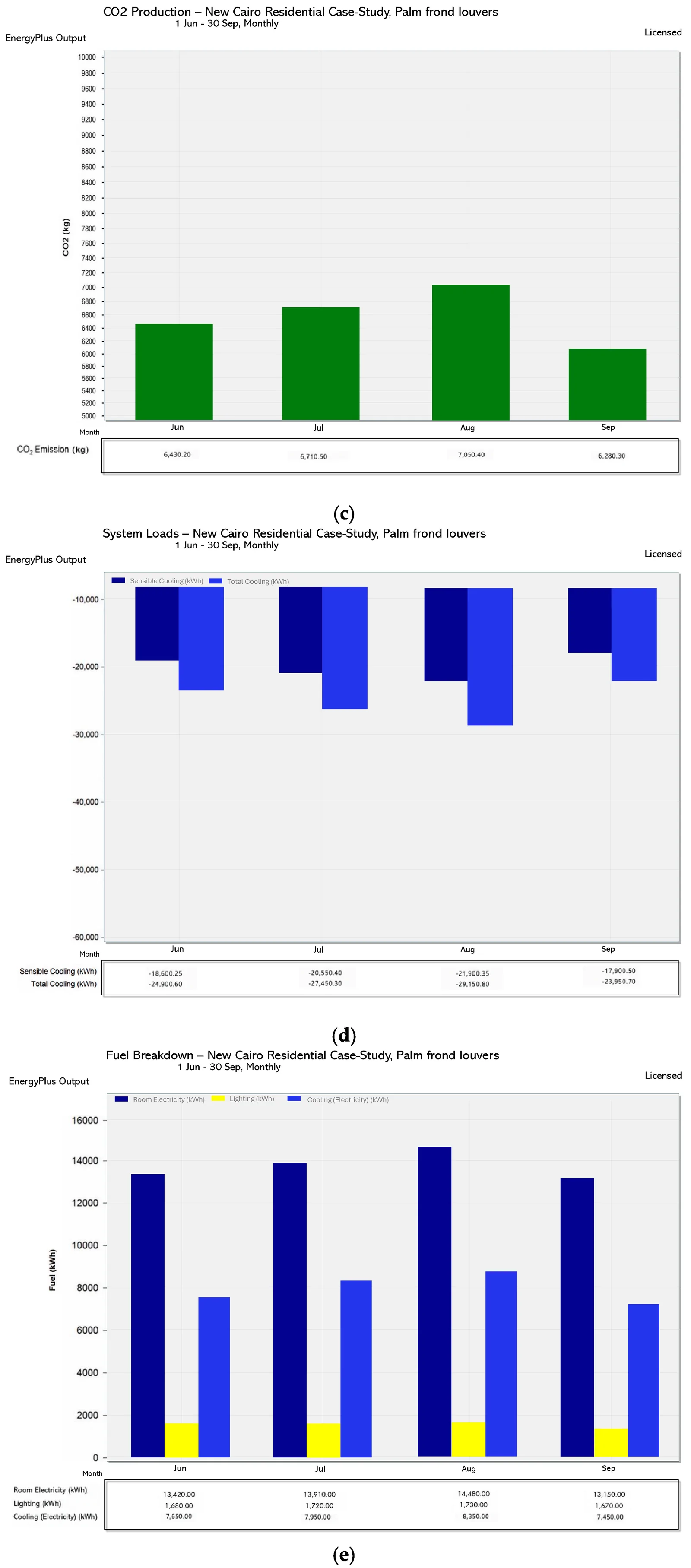
(**a**) indoor thermal comfort and temperature indicators. (**b**) internal and solar heat gains and total latent load. (**c**) operational CO_2_ emissions. (**d**) sensible and total cooling loads. (**e**) fuel breakdown, including room electricity, lighting, and cooling electricity consumption.

## Data Availability

The original contributions presented in this study are included in the article. Further inquiries can be directed to the corresponding author.

## Abbreviations

BWh	Hot Desert Climate (Köppen–Geiger Classification)
HVAC	Heating, Ventilation, and Air Conditioning
NIR	Near-Infrared
U-value	Overall Thermal Transmittance Coefficient

## Appendix A

The thermo-physical characterization of the investigated bio-based and hybrid retrofit materials was compiled from literature and manufacturer data to ensure that the simulations reflect realistic envelope performance. The key parameters considered were bulk density, thermal conductivity, and specific heat capacity, complemented, where available, by hygrothermal indicators such as moisture content, vapor permeability, and surface radiative properties. These properties, which were used in the simulation and summarized in Table A1, show that the chosen material achieves conductivity values comparable to conventional insulation materials while also providing advantageous vapor-open and radiative behavior suited to hot-arid climates.

**Table A1:** Thermo-physical properties of the proposed bio-based and hybrid retrofit materials.

Material/Layer	Density (kg/m^3^)	Thermal Conductivity λ (W/m·K)	Specific Heat (J/kg·K)	Other Relevant Properties
Rice straw	90–150	0.045–0.055	1500–2000	Moisture content 8–15%; vapor permeability ≈ 0.3–0.4 mg/(Pa·h·m); R ≈ 1.82 m^2^K/W for a 0.10 m panel [91]
Palm fronds (dry)	180–250	0.08–0.12	~1500 (literature)	Intercept ≈ 70–80% of incident solar radiation depending on overlap and orientation [92].
Jute fabric	~1400	0.04–0.05	≈1360	High emissivity 0.85–0.90; porous woven structure [90]
Cotton-waste insulation (core layer)	40–50	0.035–0.04	≈1300	Porosity > 90%; used between reflective aluminum sheets [93,94,95]
Corn-husk fiber insulation	80–100	≈0.04	≈1400	Lightweight, porous, air-entrapping fibers [93]
Aluminum sheets (hybrid cladding)	2700	≈205	≈900	Solar reflectance 0.75–0.85; emissivity 0.05–0.10 [96]

## Appendix B

The main input parameters and operational assumptions used in the Design Builder/Energy Plus simulation model are presented in this Appendix B in Table A2, aiming to increase the transparency and reproducibility of the present study. It includes the primary operational parameters used in the simulations, such as occupancy, lighting, equipment schedules, HVAC operation, and other relevant modeling assumptions.

**Table A2:** Main Design Builder/Energy Plus Simulation Input Parameters.

Parameter	Value
Simulation period	June–September
Occupancy	4–5 occupants per apartment
Occupancy schedule	Residential schedule (Design Builder default)
Lighting schedule	Residential schedule (Design Builder default)
Equipment schedule	Residential schedule (Design Builder default)
HVAC system	Split air-conditioning units
Cooling setpoint	22 °C
HVAC operation	During occupied hours
Natural ventilation	Disabled during cooling season

## Appendix C. Installation Concept, Cross-Sectional Details, and Alternative Operable Configurations of the Proposed Biomimetic Louver System

The conceptual sketches presented in this appendix illustrate the design development Figure A1, Figure A2, Figure A3, Figure A4, Figure A5 and Figure A6; the installation concept; structural configuration Figure A1; and potential fabrication approach of the proposed biomimetic louver retrofit system Figure A2 and Figure A3. They provide a visual representation of the louver arrangement, solar-shading strategy, daylight management concept, material composition, hybrid panel assembly, and alternative operable configurations, including fixed, sliding, folding, and perforated louver systems. The sketches also demonstrate the conceptual evolution from a fully closed louver configuration (Type A) to a perforated sliding system with micro-apertures (Type B) in Figure A4, Figure A5 and Figure A6, inspired by passive thermoregulatory strategies observed in nature.

These illustrations were prepared by the authors to support the interpretation of the proposed retrofit concept and to clarify the conceptual construction and installation of the investigated louver alternatives. They are intended solely for illustrative purposes and do not represent the exact Design Builder simulation models used in numerical analysis. Instead, the simulations were performed using the simplified louver geometry described in Section 4, while the sketches demonstrate the broader design concept and potential implementation of the proposed biomimetic louver system.

## Appendix D. Contour Plots in Design Builder

This appendix includes representative contour plots from the Design Builder simulation model for the base case and the biomimetic louver retrofit scenarios explored, as shown in figures listed in Appendix D.1, Appendix D.2, Appendix D.3, Appendix D.4, Appendix D.5, Appendix D.6 and Appendix D.7. The visualization results complement the quantitative results reported in the main manuscript by providing visual representations of the simulated thermal performance. For the sake of conciseness and readability of the main text, the visualization outputs are provided here for completeness.
